# Metagenomic Profiling Reveals Lignocellulose Degrading System in a Microbial Community Associated with a Wood-Feeding Beetle

**DOI:** 10.1371/journal.pone.0073827

**Published:** 2013-09-04

**Authors:** Erin D. Scully, Scott M. Geib, Kelli Hoover, Ming Tien, Susannah G. Tringe, Kerrie W. Barry, Tijana Glavina del Rio, Mansi Chovatia, Joshua R. Herr, John E. Carlson

**Affiliations:** 1 Intercollege Graduate Program in Genetics, Huck Institutes of the Life Sciences, The Pennsylvania State University, University Park, Pennsylvania, United States of America; 2 Tropical Crop and Commodity Protection Research Unit, United States Department of Agriculture Agriculture Research Service Pacific Basin Agricultural Research Center, Hilo, Hawaii, United States of America; 3 Department of Entomology and Center for Chemical Ecology, The Pennsylvania State University, University Park, Pennsylvania, United States of America; 4 Department of Biochemistry and Molecular Biology, The Pennsylvania State University, University Park, Pennsylvania, United States of America; 5 Department of Energy (DOE) Joint Genome Institute, Walnut Creek, California, United States of America; 6 Intercollege Graduate Program in Plant Biology, Huck Institutes of the Life Sciences, The Pennsylvania State University, University Park, Pennsylvania, United States of America; 7 The Schatz Center for Tree Molecular Genetics, Department of Ecosystem Science and Management, The Pennsylvania State University, University Park, Pennsylvania, United States of America; 8 Department of Bioenergy Science and Technology, Chonnam National University, Gwangju, South Korea; Universidade Federal do Rio de Janeiro, Brazil

## Abstract

The Asian longhorned beetle ( *Anoplophoraglabripennis* ) is an invasive, wood-boring pest that thrives in the heartwood of deciduous tree species. A large impediment faced by *A* *. glabripennis* as it feeds on woody tissue is lignin, a highly recalcitrant biopolymer that reduces access to sugars and other nutrients locked in cellulose and hemicellulose. We previously demonstrated that lignin, cellulose, and hemicellulose are actively deconstructed in the beetle gut and that the gut harbors an assemblage of microbes hypothesized to make significant contributions to these processes. While lignin degrading mechanisms have been well characterized in pure cultures of white rot basidiomycetes, little is known about such processes in microbial communities associated with wood-feeding insects. The goals of this study were to develop a taxonomic and functional profile of a gut community derived from an invasive population of larval *A* *. glabripennis* collected from infested host trees and to identify genes that could be relevant for the digestion of woody tissue and nutrient acquisition. To accomplish this goal, we taxonomically and functionally characterized the *A* *. glabripennis* midgut microbiota through amplicon and shotgun metagenome sequencing and conducted a large-scale comparison with the metagenomes from a variety of other herbivore-associated communities. This analysis distinguished the *A*. *glabripennis* larval gut metagenome from the gut communities of other herbivores, including previously sequenced termite hindgut metagenomes. Genes encoding enzymes were identified in the *A* *. glabripennis* gut metagenome that could have key roles in woody tissue digestion including candidate lignin degrading genes (laccases, dye-decolorizing peroxidases, novel peroxidases and β-etherases), 36 families of glycoside hydrolases (such as cellulases and xylanases), and genes that could facilitate nutrient recovery, essential nutrient synthesis, and detoxification. This community could serve as a reservoir of novel enzymes to enhance industrial cellulosic biofuels production or targets for novel control methods for this invasive and highly destructive insect.

## Introduction

Cellulose and hemicellulose represent some of the most abundant, renewable carbohydrate resources on the planet, comprising the largest natural source of fermentable sugars, which could be utilized for ethanolic biofuel production [1]. Despite the abundance of these polysaccharides, a major impediment to accessing fermentable sugars from these carbohydrates for large-scale industrial ethanol production is the presence of lignin [2], a stereotypically irregular, aromatic biopolymer comprised of phenylpropanoid aryl alcohol subunits and articulated by over 12 types of chemical bonds [3]. Highly resilient β-aryl ether and carbon-carbon bonds constitute the majority of the linkages in hardwood lignin, which are resistant to hydrolysis and difficult to disrupt. However, wood-feeding insects, in collaboration with their gut microbial communities, have the capacity to produce enzymes that facilitate the degradation of lignocellulosic material [4,5]. Accordingly, these microbial communities constitute unique ecosystems that may serve as reservoirs of novel proteins and enzymes that could be exploited to enhance the efficiency of industrial biomass pre-treatment processes, decoupling lignin from wood polysaccharides and facilitating access to fermentable sugars in cellulose and hemicellulose. Of recent interest is the gut community of *Anoplophoraglabripennis* [Order Coleoptera; Family Cerambycidae], an invasive, xylophagous beetle that colonizes and feeds in a broad range of apparently healthy tree species, including several genera commonly planted as short rotation biofeedstocks (e.g., *Populus* and *Salix*) [6,7]. A large community of microbes capable of producing cellulolytic and hemicellulolytic enzymes in the *A* *. glabripennis* midgut was previously described [8,9]. Analysis of *A* *. glabripennis* frass also revealed the presence of lignin degradation products [8], suggesting that its gut microbial community or the insect itself also harbors lignin degrading genes. The most dominant modification to lignin detected in *A* *. glabripennis* was propyl side chain oxidation, a reaction associated with white rot fungal lignin degradation that is not known to be catalyzed by bacterial- or animal-derived enzymes [10]. White rot fungal isolates have not been previously detected in association with *A* *. glabripennis* using either culture-dependent or culture-independent approaches [9,11–13], suggesting that the lignin-degrading capacity of this system is unique from well-characterized, pure-culture canonical fungal systems. Therefore, the assemblage of microbes associated with the *A* *. glabripennis* midgut represents an excellent candidate for mining novel lignocellulose degrading enzymes for biofuel applications.

Many members of the family Cerambycidae, including *A* *. glabripennis* , produce their own endogenous cellulases (endoglucanases and β-glucosidases) and other plant cell wall degrading enzymes [9,14–16]. However, interaction with microbes has been observed to enhance cellulase activities and is hypothesized to enhance glucose release from cellulose in the guts of several beetle species, including *A* *. glabripennis* [17]. For example, disruption of the gut microbiota induced by feeding on a cellulose-based artificial diet containing bacteriostatic and fungistatic agents results in a tangible reduction in cellulase complex activity (endoglucanases, exoglucanases, and β-glucosidases) in the *A* *. glabripennis* midgut [9]. In addition, insects and other herbivores are generally not capable of producing a full arsenal of O-acetylglucuronxylan-degrading enzymes and they are also generally unable to utilize pentose sugars present in xylan (e.g., D-xylose) without the aid of xylose-degrading microbes [18]. Although animal-derived enzymes have been hypothesized to be involved in lignin degradation [19] and an endogenous termite laccase can chemically modify lignin alkali and degrade lignin phenolics *in vitro* [20], microbes living in the guts of wood-feeding insects also have the capacity to produce enzymes that contribute to or enhance endogenous ligninase activities supplied by host enzymes [21,22]. Therefore, herbivorous animals, and specifically wood-feeding insects, likely benefit from enzymes produced by microbes to facilitate the digestion of woody tissue.

Wood-feeding insects exploit a variety of strategies to liberate carbohydrates from recalcitrant plant tissues and most wood-feeding insects maintain obligate associations with microbes. Associations of microbes with wood-feeding insects occur through cultivation of wood-degrading fungi [23], direct ingestion of fungal or bacterial enzymes [17], preferential feeding on compromised (stressed/decaying) trees whose structural polysaccharides have been previously disrupted by environmental wood-degrading microbes [24], or endosymbiosis with wood-degrading microbes [25]. These microbial affiliates are thought to make important contributions to lignocellulose digestion in a phylogenetically diverse array of insects, including several beetle species where microbial fermentation products have been detected in the gut [26]. Despite the associations between wood-feeding insects and microbes, the fate of lignin and the lignin degrading abilities of the microbial communities associated with many wood-feeding insects (with the exception of termites) [27] are largely uncharacterized; furthermore, no lignin degrading genes or proteins outside the white rot basidiomycetes have been annotated in metagenomes sampled from any wood-feeding insect microbial communities to date.

Wood-boring cerambycids harbor large communities of microbes, but little is known about their metabolic potential, other than the role of yeast-like gut symbionts in the digestion of hemicellulose and fermentation of xylose, which has been extensively studied [28]. Community profiling of wood-feeding cerambycid guts has revealed a striking degree of diversity in terms of community richness. In general, stenophagous insects with restricted host ranges tend to have less complex and more static gut communities than polyphagous wood-feeding insects that can colonize a broad range of host tree species and tend to have more diverse and plastic communities. This diversity and plasticity is hypothesized to allow these insects to colonize and thrive in a broader range of host trees [11]. Microbial community profiling of *A* *. glabripennis* larvae feeding in a variety of host tree species demonstrated that the composition of the community was plastic and varied by host tree species [9]. However, the composition of the *A* *. glabripennis* midgut bacterial community was distinct from the wood bacterial community sampled from unforaged sections of the tree [12]. Also, members of the *Fusarium* *solani* species complex 6 (group FSSC-6) have been consistently detected in the midguts of *A* *. glabripennis* larvae collected from multiple geographic locations and multiple host tree species, as well as larvae feeding on sterilized artificial diet [13]. These findings suggest that not all of the microbes detected in the gut are acquired directly from the host tree.

The primary goals of this study were to provide a functional and taxonomic profile of the larval midgut microbial community of an invasive *A* *. glabripennis* population feeding on a preferred host (silver maple; *Acer* *saccharinum* ) through next generation sequencing of small ribosomal subunit (SSU) amplicons and total DNA collected from the *A* *. glabripennis* midgut microbiota. Through this analysis, we compiled a suite of candidate genes found in the *A* *. glabripennis* microbial community whose annotations are consistent with lignin-, cellulose-, and hemicellulose-degrading capabilities and other genes that may have roles in nutrient synthesis and detoxification. These microbial genes are hypothesized to make key contributions to the ability of this insect to attack and develop in a broad range of healthy host trees [29,30]. We used a large-scale comparative metagenomic approach that included metagenomes derived from herbivore communities, ranging from grass-feeding ruminants to insects that thrive on highly complex woody substrates, to demonstrate that the *A* *. glabripennis* midgut metagenome was distinct from other host-associated metagenomes and could thus provide valuable insights into the interactions between wood-feeding beetles and their microbial affiliates that contribute to the digestion of woody tissue.

## Methods

### Preparation of Insect Cell Free DNA for Community Profiling and Shotgun Sequencing

Five fourth instar *A* *. glabripennis* larvae actively feeding in the heartwood of a preferred host tree ( *Acer* *saccharinum* ; silver maple) were collected from a field site located in Worcester, MA and were transported under permit conditions to a USDA-approved quarantine facility at The Pennsylvania State University for dissection and processing. The sample collection was conducted at a field site that was part of a United States Department of Agriculture’s eradication effort. Permission by the United States Department of Agriculture and by local authorities was obtained under the general permit (P526P-12-02646) Insects were sterilized twice in 70% ethanol to remove surface-contaminating microbes and residual ethanol was removed with a single rinse in sterile milliQ water. Insects were dissected and guts were removed under sterile conditions. For this experiment, we chose to focus exclusively on microbes associated with the midgut contents since this is the most prominent region in the guts of cerambycids. To enrich the sample for microbial cells and exclude insect tissue, the insect-derived peritrophic matrix (PM) that surrounds and protects the food bolus was separated from the midgut contents and DNA was extracted from microbes adhering to the food. DNA was extracted using the Fast DNA Spin Kit for Soil (MP Biomedicals, Santa Ana, CA), which was chosen due to its abilities to lyse cell walls from a variety of microbes and remove plant polysaccharides and other plant secondary metabolites that can co-extract with DNA and interfere with downstream processes. DNA was quantified using a Nano Drop 1000 spectrophotometer (Thermo-Scientific, Walthan, MA) and approximately 1 µg of DNA was used for 16S/18S amplicon and shotgun (total DNA) 454 library construction (Roche, Branford, CT).

### 454 Amplicon Pyrosequencing to Taxonomically Identify Microbes Associated with the A glabripennis Midgut

To identify the bacterial and fungal taxa found in association with the *A* *. glabripennis* midgut and to confirm that this sample was successfully enriched for microbial DNA prior to shotgun sequencing, a 16S/18S amplicon library encompassing the V6-V8 hypervariable regions was constructed using a set of primers designed to co-amplify both 16S bacterial rDNA and 18S fungal, insect, and plant rDNA from positions 926F to 1392R [31]. The amplicon library was constructed following the Department of Energy-Joint Genome Institute’s Standard Operating Procedure. In brief, 20 ng of genomic DNA were added to a PCR cocktail containing 6 µL 5X PCR buffer, 2 µL GC melt solution (Clonetech, Mountain View, CA), 0.4 µL Taq Polymerase (Advantage 2 Polymerase, Clonetech, Mountain View, CA), 0.4 µL 10 mM dNTPs (Fermentas, Pittsburgh, PA), 1 µL 25 nM forward primer (926F: 5’-*CCTATCGGGTGTGTGCCTTGG*CAGTCTCAGAAACTYAAAKGAATTGACGG-3’) and 1 µL 25 nM reverse primer (1392R: 5’-CCATCTCATCCCTGCGTGTCTCCGACTCAG**CTACT**ACGGGCGGTGTGTGC-3’). GC melt solution (Clonetech, Mountain View, CA) and Advantage 2 Polymerase (Clonetech, Mountain View, CA) were used to improve amplification efficiency of templates with high GC content. Primers were constructed using the standard 454 Titanium adaptor sequence (italics) and a five base-pair bar code incorporated into the reverse primer (bold). PCR thermal cycling conditions included an initial denaturation for three minutes at 95°C followed by 25 cycles of 95°C for 30s, 50°C for 45s, and 72°C for 90s and a final extension at 68 °C for 10 minutes. Product quality was assessed by agarose gel electrophoresis and the final product was purified using SPRI beads and quantified using the Quant-IT dsDNA Assay on a Qubit fluorimeter (Life Technologies, Carlsbad, CA). Approximately 7,000 reads were sequenced using 454 Titanium chemistry (Roche, Branford, CT). High quality reads greater than 250 bp in length were clustered into operational taxonomic units (OTUs) at 97% similarity and rarefaction curves and richness estimates were computed using the program mothur (version 1.2.22) [32]. Putative chimeras were identified using UCHIME [33] and were omitted from the analyses. Sequences for representative OTUs were compared to the non-redundant nucleotide database using BLASTN (BLAST-2.2.23) [34] with an e-value threshold of 0.00001 to determine whether the OTU was of bacterial, fungal, insect, or plant origin. Bacterial reads were classified using Ribosomal Database Project (RDP) Classifier [35], with an 80% confidence threshold for taxonomic classifications; sequences classified as mitochondrial or chloroplast in origin were omitted from the analysis. Fungal reads were classified by comparison to the non-redundant nucleotide database using BLASTN (BLAST-2.2.23) with an e-value threshold of 0.00001 followed by MEGAN classification [36] of the top ten blast alignments using the least common ancestor algorithm. Alignments to unidentified or uncultured fungi were removed from BLAST results prior to MEGAN classification. Plant- and insect-derived OTUs were excluded from the analysis. Representative sequences of each bacterial OTU were aligned with ClustalW and were trimmed to 250 bp in length for phylogenetic reconstruction using Garli (version 2.0) [37]. TIM1 + I + G was chosen as the optimal evolutionary model by jModelTest [38] and 500 bootstrap replicates were compiled to generate a consensus tree. High quality 454 amplicon reads are deposited in the NCBI Sequence Read Archive (SRA) under the accession number SRR767751.

### Phylogenetic Binning and Functional Analysis of A glabripennis Midgut Microbiota Using Shotgun 454 Pyrosequencing

454 shotgun libraries were constructed using a modified version of the 454 standard library protocol. In brief, 500 ng of DNA were sheared using a sonicator (Covaris, Woburn, MA) and fragments ranging from 500 to 800 bp were size selected using ampure beads. DNA fragments were end-polished, purified, and ligated to 454 Titanium adapters. A fill-in reaction was performed and the ssDNA template was isolated, purified, and prepared for emulsion PCR (emPCR). Additional cycles were added to the emPCR protocol to linearly amplify 454 adapter-ligated DNA from low yield DNA extractions. A previous study comparing metagenome libraries prepared with additional emPCR cycles to libraries prepared with standard numbers of emPCR cycles revealed no substantial amplification biases in libraries prepared with extra emPCR cycles (unpublished data). Based on this study, we suspect that no major biases were introduced using this approach. A total of 1.25 million shotgun reads (382 Mb) were sequenced at the DOE-Joint Genome Institute using 454 Titanium chemistry (Roche, Branford, CT). Raw reads are deposited in the NCBI Sequence Read Archive under the accession number SRR767751.

Initially, reads were assembled using Newbler (Roche, Branford, CT), but the midgut community was diverse, containing 166 bacterial OTUs and 7 fungal OTUs and the sequencing depth per OTU was too low to generate a high quality assembly. Consequently, the N50 contig length was low (< 1000 bp), and coverage across contigs was not uniform. There was also significant possibility of generating chimeric contigs consisting of reads from more than one bacterial taxon (Table 1) [39]. We felt the slight improvement in contig sequence length versus raw read length was outweighed by these assembly issues; therefore, rather than using assembled contigs, high quality shotgun reads were treated as individual gene tags, which were used for annotations (with the exception of comparisons to other metagenome communities and candidate lignin degrading gene comparisons, in which assembled contigs were used to maintain consistency with the other datasets). For annotation and analysis of the unassembled reads, low quality reads with mean quality scores below 20, reads containing repetitive regions, and reads less than 150 bp in length were excluded from the dataset. Tags originating from non-coding RNAs, including tRNAs and rRNAs, were detected with tRNA-Scan [40] and HMMer using HMM profiles for prokaryotic, eukaryotic, and archaeal small subunit and large subunit rRNAs [41,42]. While tRNAs were filtered out of the dataset and were not utilized in downstream functional analyses, small subunit (16S and 18S) rRNAs detected were taxonomically classified by alignment to the SILVA SSU database [43] to detect additional bacterial and fungal taxa that may not have been detected with 454 amplicon analysis due to primer inefficiencies or biases. After filtering and removing non-coding RNAs, 1.06 million reads, ranging in length from 150 to 1050 bp, remained (mean read length: 350 bp).

**Table 1:** Summary of Newbler metagenome assembly metrics.

Number of 454 Shotgun Reads Produced	1,258,810
Number of Contigs	25,838
Number of Singleton Reads	585,749
Minimum Contig Length (bp)	200
Maximum Contig Length (bp)	30,393
N20 (bp)	2,081
N50 (bp)	938
N80 (bp)	555
Total Number of Assembled (bp)	22,220,287
Total Number of Unassembled (bp)	179,346,064

454 library adapters and low quality ends were trimmed from the remaining reads. Individual reads were annotated by BLASTX comparisons to the non-redundant (NR) protein database [34] using an e-value cutoff of 0.00001 and were taxonomically classified using MEGAN (MEtaGenome ANalyzer) [36] least common ancestor classification based on the top 10 BLAST alignments for each read. Reads predicted to originate from bacterial or fungal taxa were also uploaded to the MG-RAST server [44] for gene prediction and assignment to SEED subsystems. Reads were also functionally categorized via an RPS-BLAST comparison [45] to the Clusters of Orthologous Gene (COG) database [46]. Reads were also assigned to Gene Ontology (GO) terms [47] and classified to KEGG enzyme classes [48] using BLAST2GO [49]; furthermore, reconstruction of metabolic pathways was conducted using MinPath (Minimal set of Pathways) parsimony analysis [50] of KEGG Orthology (KO) assignments. BLAST results were corroborated by 6-frame translation followed by functional domain analysis using HmmSearch [41] to scan for Pfam A domains [51]. CAZyme (Carbohydrate active enzyme) [52] carbohydrase family classifications are based on Pfam domain assignments.

### Comparisons to Other Herbivore-Related Metagenomes

Pfam domains from the *A* *. glabripennis* metagenome assembly (contigs and un-assembled singleton reads) were compared to domains from assembled (contigs and unassembled singletons) metagenome data sampled from communities associated with herbivores feeding on a diversity of plants that varied in carbohydrate and lignin composition. Pfam functional domains were chosen for comparative analysis because they are relatively short in length, which increases the likelihood that they will be correctly identified in single sequence reads. Therefore, detection and subsequent annotation of these domains are less likely to be influenced by assembly contiguity, which varied between the metagenome libraries. Annotated Pfam domains were obtained from the JGI IGM/M database for microbial communities associated with 1) **herbivores that feed on a variety of plant tissues**: panda, reindeer, honey bee, attine ant fungal garden, and wallaby; 2) **insects that feed only on phloem and/or xylem tissue**: *Dendroctonous* *frontalis* galleries and guts, *Dendroctonousponderosae* galleries and guts, *Xyleborus* *affinis* galleries and guts (larval and adult); and 3) **insects that feed only in woody tissue**: *Amitermes* *wheeleri* hindgut*,* *Nasutitermes* *sp.* hindgut*,* *Sirexnoctilio* fungal gallery, and a community affiliated with *Trichonympha* protist symbionts of termites collected from Los Padres National Forest, CA. The Pfam compositions of these communities were compared to the Pfam composition of the *Anoplophoraglabripennis* midgut community. For each community, data were normalized by total number of Pfam domains detected, weighted by contig depth when assembly information was available, and a compositional dissimilarity matrix was constructed based on Euclidean distance. For unassembled singleton reads, a contig depth of one was assumed. Samples were subjected to cluster analysis using Ward’s method. Further, the standardized data were also analyzed using unconstrained Principal Components Analysis to plot samples in multidimensional space. PCA ordination was selected because the data were determined to be linear by detrended correspondence analysis (DCA) (Beta diversity <4). Partially constrained redundancy analysis (RDA), removing effects of library size, did not significantly change the ordination, indicating that differences in library sizes do not significantly influence the ordination. All multivariate comparisons and ordinations were performed using the R statistical package with ‘vegan’ and ‘cluster’ libraries.

## Results and Discussion

### Taxonomic Classification of OTUs and Shotgun Reads

Approximately 6.7% of the total shotgun reads were classified to class Hexapoda while approximately 0.2% of the total shotgun reads were classified as plant, indicating that the metagenome library was comprised predominantly of microbial DNA. Amplicon sequencing identified seven distinct fungal OTUs and 166 bacterial OTUs using a 97% similarity threshold in mothur, while only a single insect OTU (2% of the total amplicons) and a single plant OTU (0.53% of the total amplicons) were detected. Overall, fungal reads outnumbered bacterial reads, which could be attributed to a higher relative abundance of fungal taxa in the midgut or to preferential amplification of fungal amplicons with the 926F/1392R primers used in this study, as this dominance is not reflected in the shotgun sequencing data.

**Figure 1:**
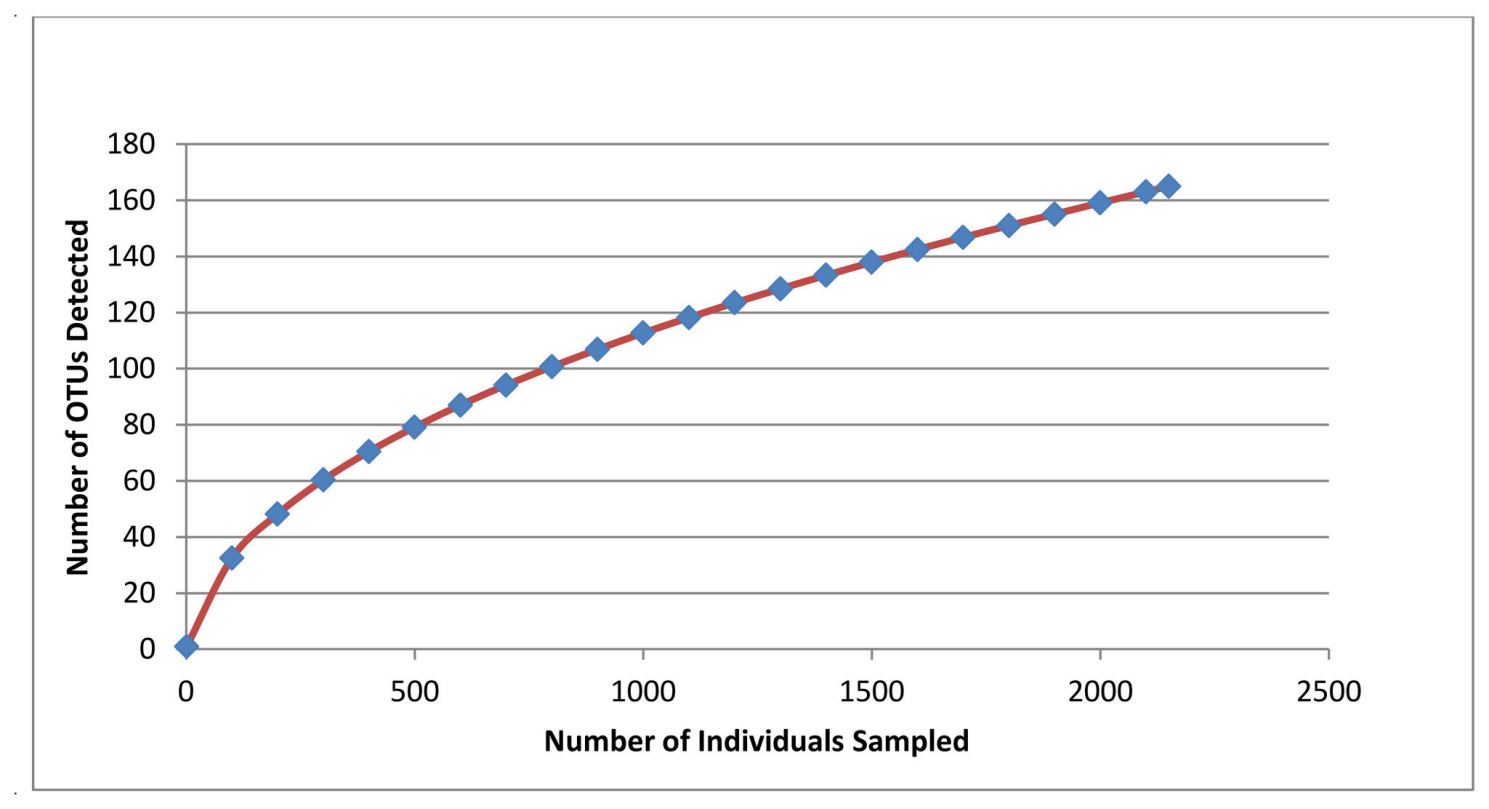
Rarefaction, richness, and diversity analyses of 16s amplicon data. Approximately 166 bacterial OTUs were detected through amplicon sequencing. Various community richness estimators consistently predicted the presence of over 300 OTUs in association with the *A* *. glabripennis* gut and, in agreement with this observation, the rarefaction curve failed to reach saturation. This indicates that additional OTUs would likely be detected with additional amplicon sequencing.

**Figure 2:**
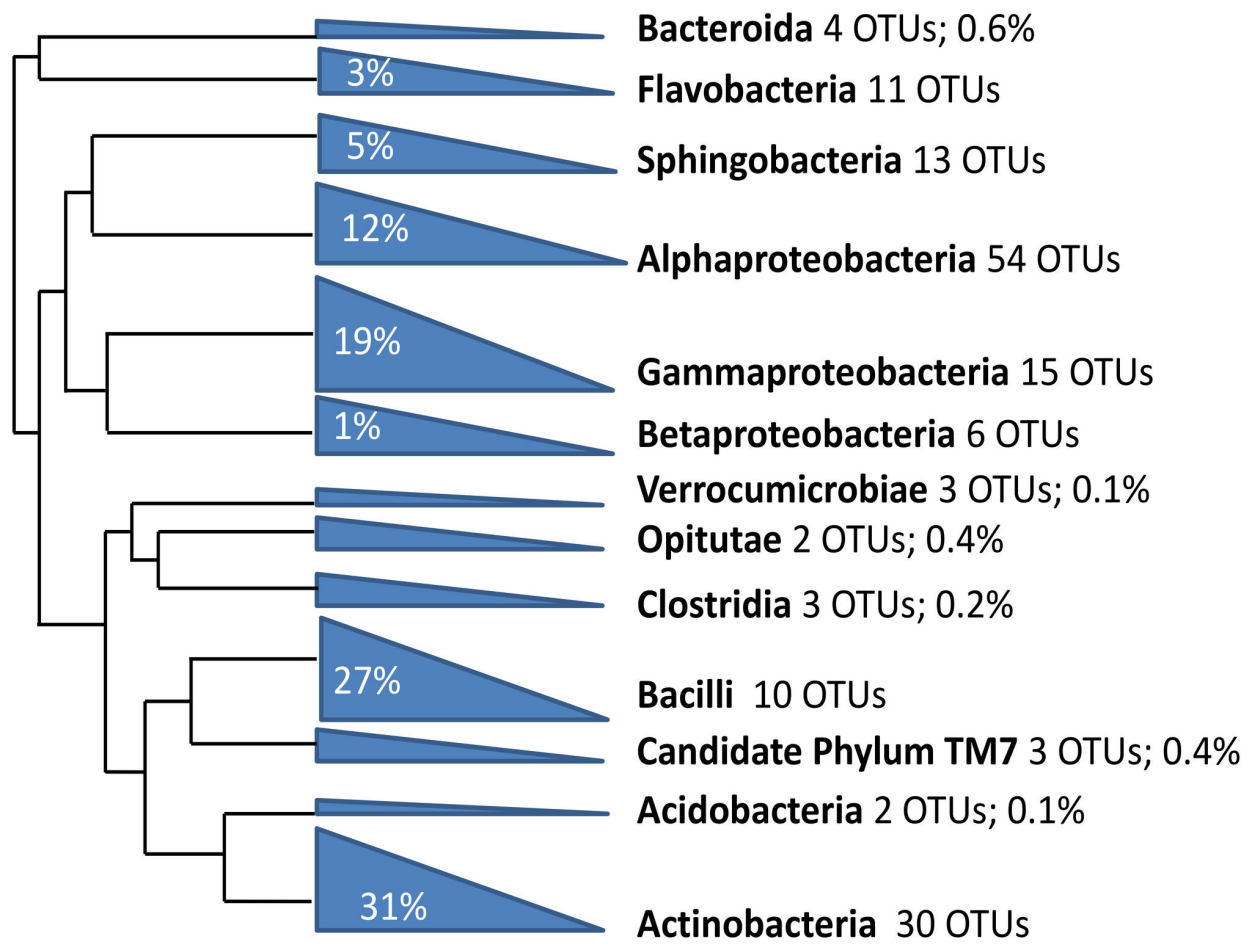
Maximum likelihood analysis of representative sequences from operational taxonomic unit analysis (OTU) of bacterial 16S rRNA amplicons. Representative sequences from each bacterial OTU were aligned with MEGA 4.0 and phylogenetic analysis using was performed using GARLI 2.0 (500 bootstrap pseudoreplicates and TIM1+I+G evolutionary model). Nodes were collapsed and labeled by taxonomic class. Number of OTUs and percentage of amplicons assigned to each class are labeled. OTUs that could not be assigned to class level by RDP were omitted from the analysis.

OTU taxonomic classification with RDP classifier detected the presence of 166 OTUs in seven bacterial phyla in the midgut community including Actinobacteria (30 OTUs), Bacteroidetes (29 OTUs), Chlamydiae (1 OTU), Firmicutes (14 OTUs), Proteobacteria (80 OTUs), candidate phylum TM7 (3 OTUs), and Verrucomicrobia (5 OTUs), while four OTUs could not be conclusively assigned to any previously-characterized bacterial phyla. Rarefaction analysis and Chao richness estimates predict the presence of over 350 bacterial OTUs (95% confidence interval range: 266-517 OTUs), demonstrating that deeper sampling of amplicon data may result in the detection of additional less abundant bacterial taxa (Figure 1 and Table 2). The most taxonomically-diverse phylum in terms of OTU richness was Proteobacteria, containing 80 distinct OTUs assigned to 22 different families. At the class level, 15 different bacterial classes were identified and the midgut community was dominated by six taxonomic classes (Figure 2 and Table 3). Overall, the single most-prevalent OTU, which comprised over 21% of the bacterial amplicons, was a member of the family Leuconostocaceae that could not be classified to genus level by RDP. Comparison of this OTU to 16S sequences curated in the RDP database revealed that it had highest nucleotide sequence similarity to bacteria in the genus *Leuconostoc* . Other predominant OTUs were assigned to the family Enterobacteriaceae (8.4% bacterial amplicons), the family Microbacteriaceae (8.3% bacterial amplicons), and to the higher phylum Actinobacteria (9.3% bacterial amplicons). Many OTUs could not be definitely assigned to low taxonomic levels, suggesting that the *A* *. glabripennis* midgut microbiota may serve as a reservoir for novel microbes. With the exception of the higher overall abundance of fungal 18s OTUs relative to bacterial 16s OTUs, the results of OTU abundance and classification were corroborated by phylogenetic binning of shotgun reads, which is less impacted by amplification biases relative to PCR-based approaches (Figure S1).

**Table 2:** Species richness and diversity calculations for bacterial OTUs detected in the *A* *. glabripennis* gut.

# OTUs Observed	Chao Richness	95% CI Chao	Ace Richness	95% CI Ace	Jackknife Richness	95% CI Jackknife	Simpson Diversity (1-D)	95% CI Simpson Diversity (1-D)
166	354	266-518	437	370-526	657	434-870	0.919	0.912-0.925

**Table 3:** Species richness and diversity calculations for fungal OTUs detected in the *A* *. glabripennis* gut.

# OTUs Observed	Chao Richness	95% CI Chao	Ace Richness	95% CI Ace	Jackknife Richness	95% CI Jackknife	Simpson Diversity (1-D)	95% CI Simpson Diversity (1-D)
7	11	8-31	18	9-101	12	6-18	0.51	0.49-0.52

### Identification of Cellulose-, Hemicellulose- and Aromatic Compound- Degrading Bacterial Taxa

Several genera of bacteria were detected in the *A* *. glabripennis* midgut community that have been previously implicated in the degradation of lignocellulose, hemicellulose, and other aromatic hydrocarbons, including the following lignocellulose degrading bacteria previously isolated from the *A* *. glabripennis* midgut on carboxymethylcellulose-containing media or detected previously through 16S analyses: *Brachybacterium* , *Bradyrhizobium* , *Corynebacterium* *,* *Rhizobium* , *Pseudomonas* , *Sphingomonas* , and *Xanthamonas* [9,12]. Furthermore, the midgut community sampled for this study strongly resembles the taxonomic compositions of larval gut communities previously sampled from insects feeding in *Acer* *saccharinum* in a separate population (Brooklyn, NY) [9] and from beetles collected in China [11], suggesting a consistent relationship between these microbial taxa and *A* *. glabripennis* . Of significance is that, unlike the termite and other herbivore-associated gut communities, the microbiota associated with the *A* *. glabripennis* midgut is dominated by aerobes and facultative anaerobes with very few obligate anaerobic taxa. To date, all characterized large-scale lignin degrading reactions require oxygen and have only been demonstrated in aerobic environments [53], such as the *A* *. glabripennis* midgut [11].

### Identification of Fungal Community

**Figure 3:**
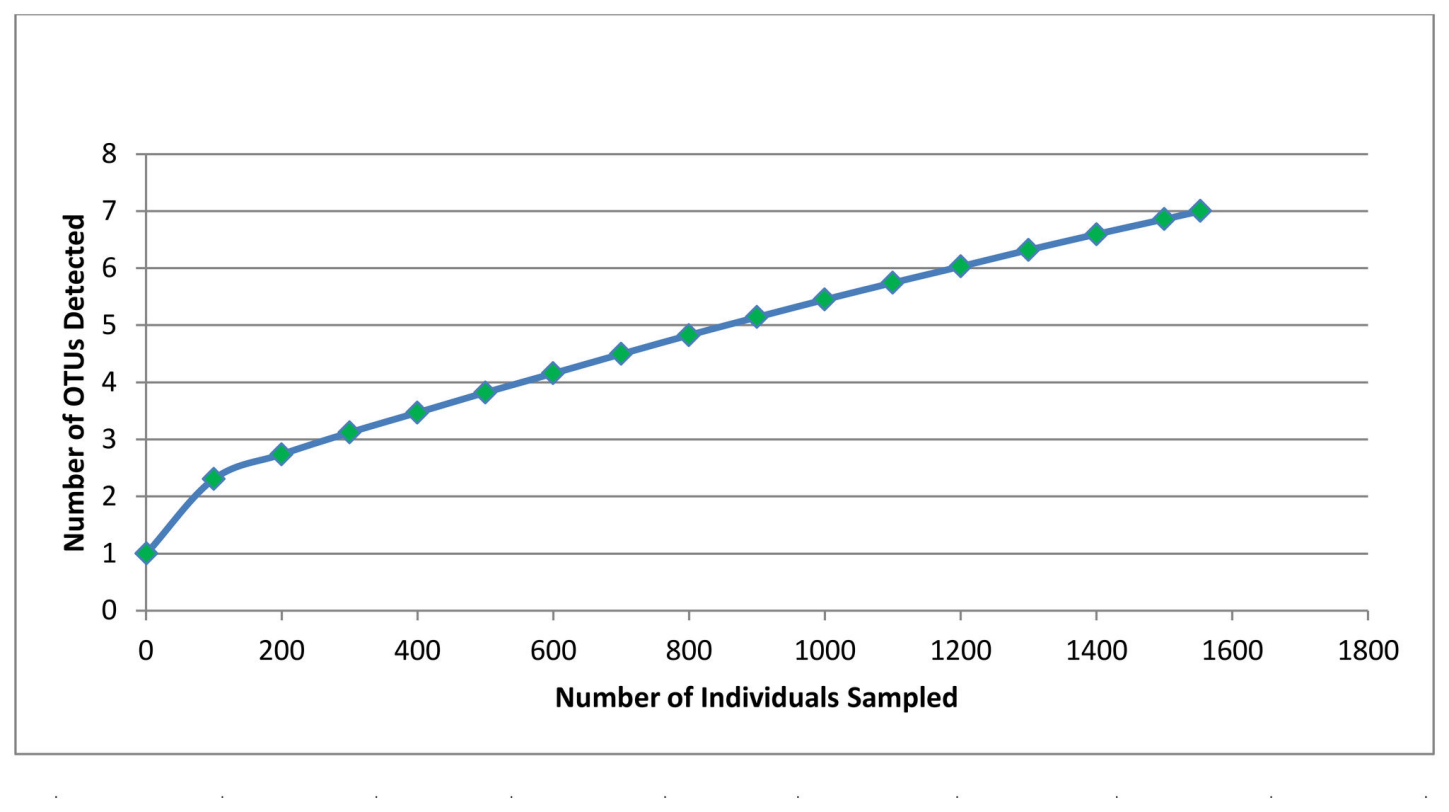
Rarefaction, richness, and diversity analyses of 18S amplicon data. Seven fungal OTUs were detected through amplicon sequencing. While rarefaction begins to approach saturation, richness estimates predict the presence of at least 11 fungal OTUs indicating that additional sampling may be necessary. This scenario is likely since additional 18S rRNAs from fungal taxa not detected in the 18S amplicons were detected in the shotgun reads (e.g., *Fusarium* spp.).

Fungi are frequently encountered in guts of wood feeding insects [54], including *A* *. glabripennis* [13]; however, in contrast to the bacterial community, the fungal community is considerably less diverse, containing approximately 7 distinct OTUs. Rarefaction analysis and richness estimates predict 18 fungal OTUs (95% confidence interval: 8-31 OTUs) (Figure 3). Compared to the 16S region in bacteria, 18S regions in fungi display considerably less sequence heterogeneity [55], even among distant relatives and an accurate assessment of fungal diversity in the *A* *. glabripennis* midgut may be underestimated. All fungal taxa detected belonged to the phylum Ascomycota, confirming a low abundance or complete absence of white-rot basidiomycetes in the midgut microbiota. All of the fungal taxa detected were yeasts assigned to the family Saccharomycetaceae. However, most could not be conclusively classified to genus level with MEGAN, but had highest-scoring BLAST alignments to the genera *Issatchenika* (3 OTUs; 58% total fungal amplicons) and *Saccharomyces* (1 OTU; 36% total fungal amplicons). The three other fungal OTUs were present as singletons and had highest-scoring BLAST alignments to the fungal genera *Geotrichum*, *Pichia*, and an unclassified member of the family Archaeosporaceae. Many of these genera are phylogenetically close relatives to yeasts isolated from the guts of other wood-feeding cerambycid beetles [56], which are often capable of processing hemicellulose and fermenting xylose into ethanol, but are not known to degrade lignin or cellulose. Many wood- and plant-feeding insects, such as leaf-cutter ants [57], wood wasps [58], bark beetles [59] and some termite species [60] maintain obligate external associations with non-yeast filamentous basidiomycete and ascomycete fungi and directly inoculate fungal isolates into their food sources, where they facilitate pre-digestion of lignocellulose and serve other nutrient-provisioning roles. These strategies substantially reduce the carbohydrate complexity and lignin content of the food substrate prior to ingestion by the insect. In contrast, *A* *. glabripennis* constitutively harbors a filamentous ascomycete belonging to the *Fusarium* *solani* species complex within its midgut [13]. Multilocus phylogenetic analysis of this isolate collected from several geographic populations revealed that the isolates harbored in the beetle gut are distinct from other previously characterized members of the *F* *. solani* species complex. Moreover, this fungus could be detected in colony-reared insects feeding on sterile diet [13], suggesting that this fungus is intricately associated with the gut. Though *F* *. solani* was not detected in the 18S fungal amplicon data, *F* *. solani* has been cultivated previously from *A* *. glabripennis* beetle guts collected at this field site [13] and reads derived from *F* *. solani* were detected in the shotgun library. This low abundance of *F* *. solani* reads in the shotgun libraries is likely due to excluding the peritrophic matrix from the sample as *F* *. solani* is likely associated with the gut wall tissue. Members of the *Fusarium* species complex are metabolically versatile and often harbor lignin peroxidase and other ligninase homologs [61], which suggests contributions to these processes in the *A* *. glabripennis* midgut [62].

### Functional Profiling of Reads Generated through 454 Shotgun Sequencing

**Figure 4:**
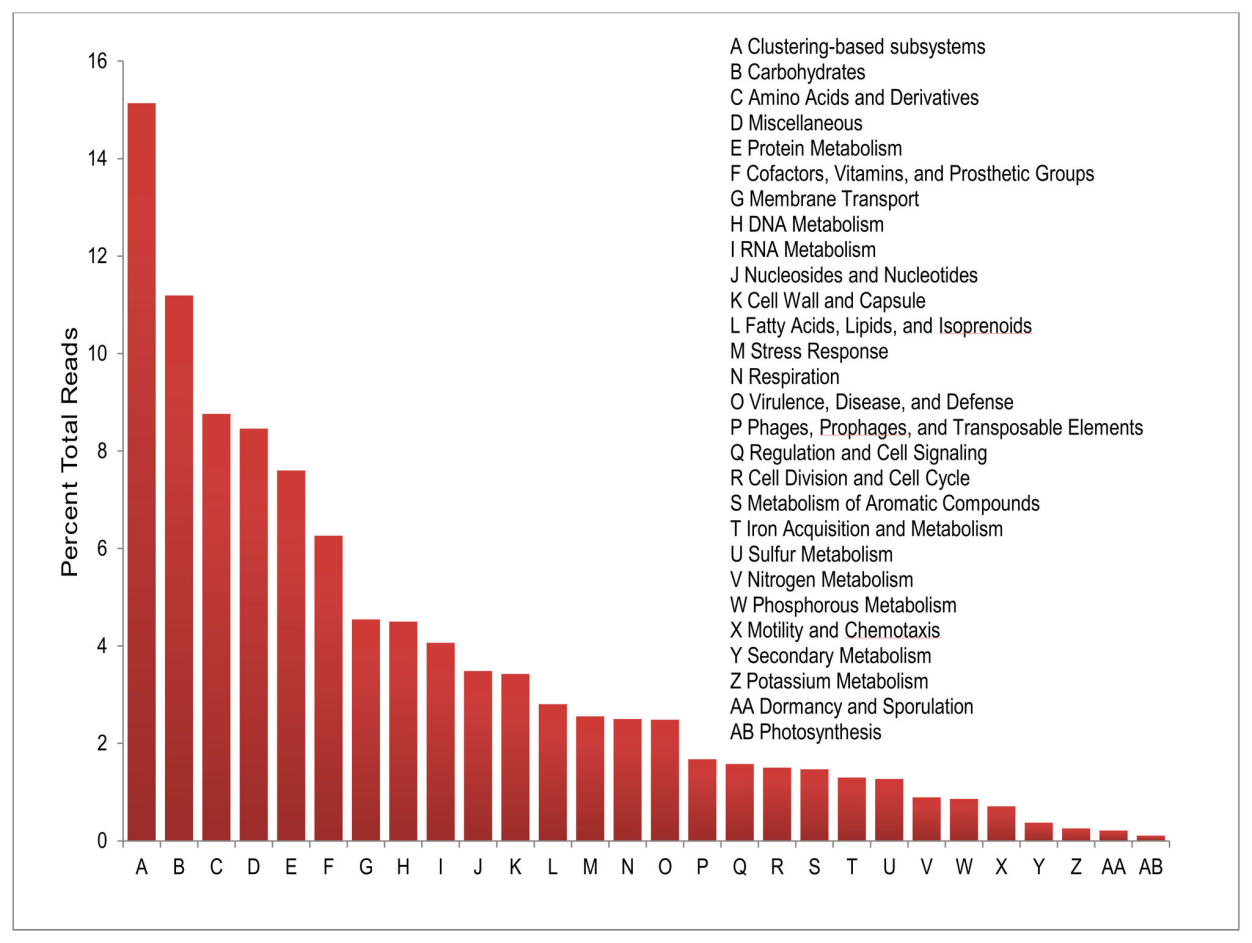
Distribution of SEED assignments generated by MG-RAST. Reads assigned to 28 SEED subsystems were detected in the *A* *. glabripennis* larval midgut metagenome. The most dominant subsystems found in association with this microbial community included clustering based subsystems, carbohydrate metabolism, and amino acid and derivatives metabolism.

Approximately 65% of the high quality 454 reads generated had BLASTX matches to proteins from the non-redundant protein database at an e-value of 0.00001 or lower. Of these reads, approximately 79% had best alignment scores to annotated proteins, while the remaining 21% had highest scoring BLAST alignments to hypothetical or uncharacterized proteins. Overall, the most abundant BLAST and Pfam domain assignments associated with the midgut microbial community belonged to ABC transporters, major facilitator transporters, alcohol dehydrogenases, and aldehyde dehydrogenases. Functional categorization of shotgun reads by both COG and SEED assignments predicted that the majority of the reads originated from pathways involved in the metabolism of carbohydrates and amino acids (Figure 4). Annotation statistics are summarized in Table 4 and annotations are publically available through MG-RAST at http://metagenomics.anl.gov/ under the identification number 4453653.3 and JGI IMG/M at http://img.jgi.doe.gov/m/ under project ID Gm00068.

**Table 4:** Summary of metagenome annotations.

**Number of High Quality Shotgun 454 Reads**	1,067,718
Number of rRNAs	397
Number of tRNAs	2,596
Number of reads with BLASTX alignments to annotated proteins in non-redundant protein database (e-value = 0.00001)	541,761
Number of reads with BLASTX alignments to hypothetical proteins in non-redundant protein database (e-value = 0.00001)	144,965
Number of reads with COG (Clusters of Orthologous Genes) assignments	357,999
Number of reads with Seed assignments	255,091
Number of reads with GO (Gene Ontology) assignments	361,412
Number of reads with KEGG assignments	173,359
Number of reads with Pfam domains	420,285
Number of reads with BLASTX alignments and Pfam domains	409,594
Number of reads with Pfam domains only (no BLASTX alignments)	10,691

### Comparison of Functional Domains from Other Herbivore Associated Microbial Communities

**Figure 5:**
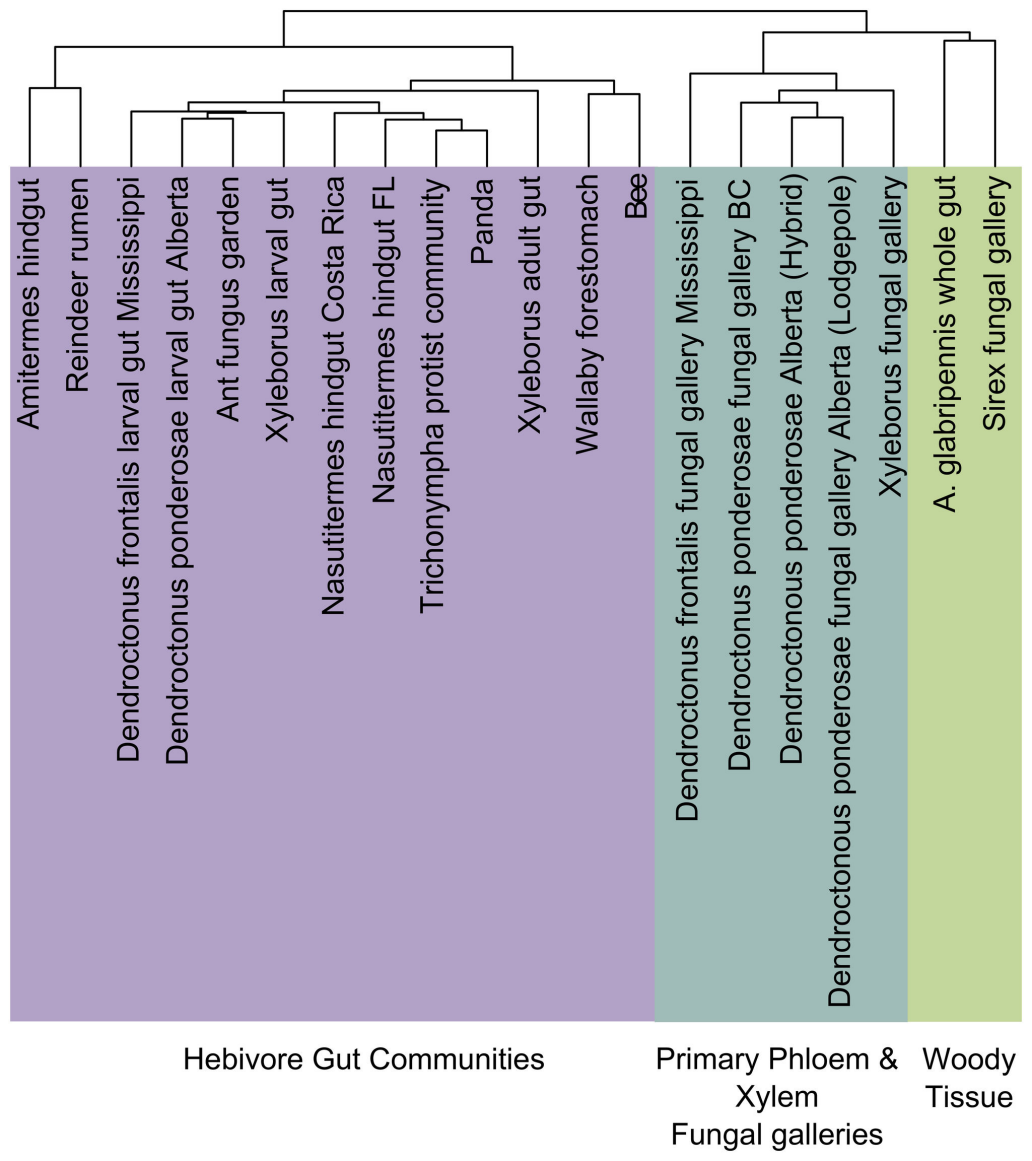
Hierarchical cluster analysis based on Pfam annotations of herbivore related metagenomes. Agglomerative hierarchical cluster analysis based on a compositional Euclidean distance matrix was conducted using Pfam annotations from various herbivore related metagenomes. Three distinct clusters representing different herbivore biome-types are highlighted and labeled. These include herbivore gut communities, fungal gallery communities associated with phoem/xylem feeding insects and communities associated with insects feeding in heartwood.

Hierarchical agglomerative cluster analysis based on Pfam abundances from herbivore-associated metagenomes did not appear to group the microbial communities based on the taxonomic relatedness of their herbivore hosts (Figure 5). Although many of the beetle gut communities and fungal gallery communities are derived from closely related beetles and cluster together, several notable exceptions suggest that factors other than taxonomic relatedness contribute to the hierarchical clustering pattern observed. For example, although *A* *. glabripennis* (Order Coleoptera) and *S* *. noctilio* (Order Hymenoptera) belong to two different insect orders, their microbial communities can be found in the same group in the cluster analysis, suggesting that they share similarities in microbial metabolic capabilities. Additionally, the two hymenopterans included in this comparison (honey bee and *Sirex*) fall into two distant clusters. However, a clear division between gut communities and fungal gallery communities is apparent, with the exceptions of the ant fungal garden, which clustered with the herbivore gut communities and was previously hypothesized to function as an external rumen [63]. The *A* *. glabripennis* midgut community is also an exception as it clustered with the fungal gallery communities. Interestingly, many of the fungal gallery communities that cluster with the *A* *. glabripennis* metagenome are hypothesized to have lignin degrading capabilities, which is in contrast to the ant fungal garden community. While cellulose and hemicellulose were preferentially degraded in the fungal gardens, lignin remained relatively unscathed and was ultimately discarded by the insects [63]. The same pattern of cell wall digestion has also been observed in the rumens of many grass-feeding herbivores [64].

Although fungal communities cultivated by bark beetles [65] are primed to synthesize nutrients and detoxify plant secondary metabolites [66], penetration of the lignin barrier enhances access to cellulose and hemicellulose present in both phloem and xylem tissues where bark beetles feed. Although the fate of lignin in the majority of these systems is unclear, lignin degradation and aromatic compound metabolism have been demonstrated in a *Fusarium* *solani* fungal gallery strain associated with xylem-feeding ambrosia beetles (e.g., *Xyleborus*) [67]. Thus, the fungal gallery communities associated with these phloem and xylem feeding beetles have the potential to harbor lignin degrading genes capable of degrading woody tissue. The final cluster in our analysis contains microbial communities associated with insects feeding on heartwood and includes the *A* *. glabripennis* midgut and *Sirex* wood wasp fungal gallery communities. Notably, these wood-feeding communities are relatively distant from those associated with the other herbivore guts or the other fungal gallery communities included in this comparison, suggesting that these communities may harbor genes that encode enzymes optimized for breaking down complex and recalcitrant woody tissue. Like *A* *. glabripennis* , the *Sirex* fungal gallery community is also capable of disrupting lignin polymers and the community contains a lignin degrading white rot fungus belonging to the genus *Amylostereum* , which produces manganese peroxidases and laccases [68].

**Figure 6:**
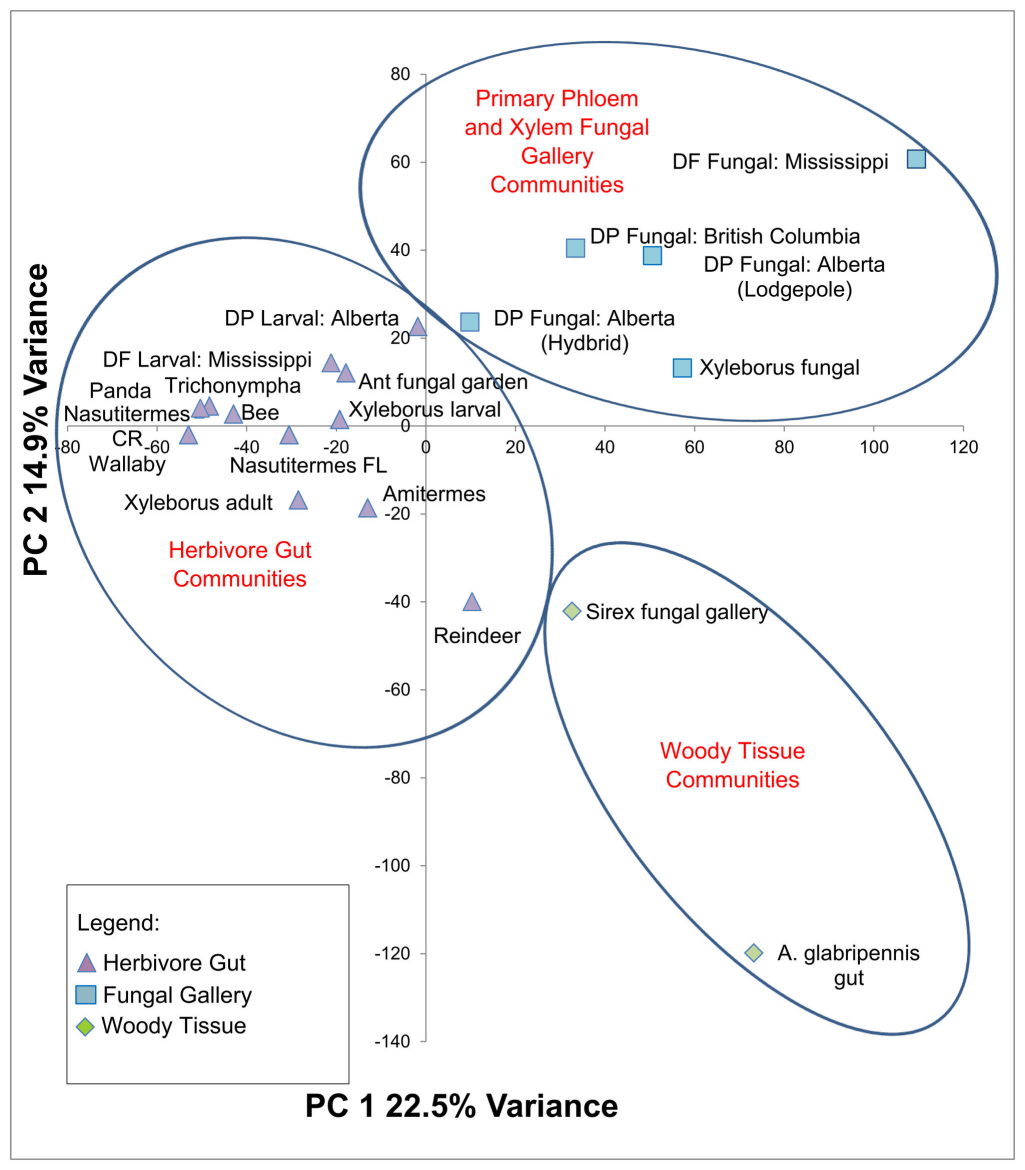
Principal components analysis (PCA) of Pfam domains from herbivore-related metagenomes. Principal components analysis was conducted to plot samples in multidimensional space. Groupings detected in agglomerative cluster analysis are preserved (Mantel test, p< 0.0001) and are color-coded by groups identified in the dendrogram. Monte Carlo Permutation Procedure (n=1000 iterations): p<0.0001 for PCA 1 and PCA2. DP: Dendroctonous ponderosae DF: Dendroctonous frontalis CR: Costa Rica, FL: Florida.

The groupings detected through hierarchical cluster analysis are also supported by Principal Components Ordination (Figure 6). The X-axis separates the majority of the gut communities from the gallery communities with the notable exception of the *A* *. glabripennis* midgut, which is clearly distinct from the other gut metagenomes and was placed in close proximity to the *Sirex* fungal gallery microbiome. The Y-axis separates fungal gallery communities associated with phloem-feeding herbivores from wood-feeding herbivores that bore deep into the heartwood. Although both *Sirex* and *A* *. glabripennis* insects feed in similar regions of their host trees, *Sirex* has a limited host range relative to *A* *. glabripennis* and feeds exclusively on the genus *Pinus* [69]. In contrast, *A* *. glabripennis* has a much broader host range and feeds in the heartwood of over 25 deciduous tree species in the United States (http://www.aphis.usda.gov/plant_health/plant_pest_info/asian_lhb/downloads/hostlist.pdf) and 47 tree species in its native range [30]. These differences in lifestyle are also reflected in the PCA ordination. Although the *A* *. glabripennis* midgut community is most similar to the *Sirex* fungal gallery community, the distance between these two metagenomes is still quite significant and could be partially driven by differences in host range breadth and environment (e.g. gut vs. gallery).

### Candidate Genes for Lignin Degrading Enzymes

Genes encoding enzymes that have been previously implicated in lignin degradation were identified in the microbiomes affiliated with both the midgut of *A* *. glabripennis* and the fungal gallery communities, and may be partially responsible for driving the grouping of these communities in the hierarchical analysis (Table S1). This is in contrast to the results of a recent comparative metagenomic study that concluded host-associated communities lacked the metabolic potential to degrade lignin [86], and may indicate that the *A* *. glabripennis* midgut community represents an exception. A number of bacterial and fungal reads with copper oxidase (Cu oxidase) Pfam domains were detected in the *A* *. glabripennis* midgut, which could have laccase-type activity *in vivo* [70]. While many of these reads had corresponding BLAST assignments to laccases, multicopper oxidases, and polyphenol oxidases, a large number of the annotations were to hypothetical proteins and could represent novel and previously uncharacterized laccase-type enzymes. While laccases do not endogenously have a high enough redox potential to cleave major linkages in polymeric lignin [71], their activity can be enhanced in the presence of natural redox mediators [72] and, they are capable of disrupting β-aryl ether bonds under these conditions. β-aryl ethers represent the most dominant linkage in hardwood lignin and as a consequence, disruption of these linkages represents a critical step in lignin degradation [73].

A number of other extracellular peroxidases that are often highly expressed by lignin degrading microbes during periods of active lignin degradation were also detected. These include iron-dependent peroxidases, thiol peroxidases, and a number of other uncharacterized peroxidases. The potential participation of these peroxidases in large-scale lignin degradation is also supported by the detection of a number of peroxide-generating enzymes containing predicted leader sequences for extracellular targeting. These included aryl alcohol oxidases, FAD oxidoreductases, glyoxal oxidases, GMC oxidoreductases, and pyranose oxidases.

Bacterial dye-decolorizing peroxidases, also known as dyp-type peroxidases, were detected in association with the *A* *. glabripennis* midgut microbiota and microbial communities associated with other wood-feeding insects, and have previously been shown to cleave β-aryl ether linkages in both syringyl and guaiacyl lignin in a hydrogen peroxide dependent manner [74]. While there is some evidence that manganese may act as a diffusible redox mediator in some bacterial dyp-type peroxidases [74], not all β-aryl ether cleaving peroxidases have identifiable manganese binding sites and thus, manganese may enhance the activity of a subset of these peroxidases [75]. Furthermore, reads for another set of β-aryl ether degrading enzymes were also discovered, which have been shown to catalyze the cleavage of these bonds in a glutathione-dependent manner. These enzymes were classified as β-etherases or glutathione-S-transferases [76]. In order to cleave β-aryl ether linkages, these enzymes first require oxidation of the C_α_ primary alcohol by aryl alcohol dehydrogenase (or C_α_ dehydrogenase) to generate a ketone group. The presence of a ketone group immediately adjacent to the ether linkage increases the polarity of the ether bond, allowing the ether bond to be easily cleaved by β-etherase, using glutathione as a hydrogen donor [77]. However, these GST (β-etherase) functional domains were not exclusively present in candidate lignin degrading genes [78] and are also associated with genes involved in detoxification (i.e., glutathione s-transferases) [79]. Therefore, only a subset of the GST domain proteins reported in this analysis are lignin degrading candidates. The role of dyp-type peroxidases and β-etherases in polymeric lignin degradation has yet to be clarified. While some bacteria harboring these genes can cleave β-aryl ether linkages in dimeric lignin model compounds and Kraft and wheat straw lignin, their ability to catalyze degradation of an intact biopolymer from woody plants is unknown [80].

Of significance is that the majority of the lignin degrading genes present in the *A* *. glabripennis* midgut community are either absent or present in very low abundances in the communities associated with herbivore guts, including, panda, reindeer, honey bee, and wallaby and termites. This finding suggests that these herbivore communities may have alternate genes and mechanisms that could have lignin degrading roles *in vivo* or that some of these gut-associated communities lack lignin degrading capabilities altogether. In contrast, these lignin degrading candidates were highly abundant in the communities associated with wood-feeding insects, including the *Sirex* fungal gallery and *A* *. glabripennis* midgut. Consistent with their hypothesized role in the pre-digestion of lignocellulose for phloem-feeding insects, many lignin-degrading candidates were also found in high abundances in the fungal galleries of phloem feeding bark beetles. Although small subsets of these lignin degrading genes were also detected in guts of phloem feeding insects, these genes are likely environmentally derived and were acquired by feeding on the fungal gallery inoculum or they may also be encoded by microbes housed in the gut. Notably, peroxidases and extracellular hydrogen-peroxide generating enzymes were overrepresented in the *A* *. glabripennis* midgut community relative to other communities included in this analysis, suggesting that this community may have alternative pathways for degrading core lignin.

Despite the high abundances of putative laccases, dyp-type peroxidases, and hydrogen peroxide generating enzymes (FAD oxidases and GMC oxidoreductases) in the fungal gallery communities and the *A* *. glabripennis* midgut community, another class of putative lignin degrading enzymes (aldo-keto reductases: AKRs) were well represented in the termite gut communities, the tamar wallaby gut community, a subset of the fungal gallery communities (e.g. *Xyleborus*, DP Fungal Alberta (hybrid), DP Fungal Alberta, and DF Fungal Mississippi), and the *A* *. glabripennis* midgut community. An endogenous termite AKR capable of degrading lignin phenolics and enhancing sugar release from pine sawdust was recently characterized [81] and subsets of microbial AKRs can act as C_α_ dehydrogenases, which can work in conjunction with β-etherases to cleave β-aryl ethers [77]. Microbial AKRs are well represented in the termite gut communities and have the potential to collaborate with host-derived AKRs to enhance ligninase activity in the gut. Interestingly, microbial AKRs are overrepresented in the *A* *. glabripennis* gut community relative to most other communities included in the comparison and have the potential to make contributions to digestion of lignin in this system. Taken together, we hypothesize that the *A* *. glabripennis* midgut metagenome has a lignin degrading capacity distinct from the termites and other herbivore associated communities that could be prospected for biotechnology purposes. This possibility is supported by the fact that biochemical modifications to lignin detected in the gut of a lower termite ( *Zootermopsis* *angusticollis* ) were different than the lignin modifications detected in the *A* *. glabripennis* gut [8].

### Candidate Genes for Cellulases and Carbohydrases

**Figure 7:**
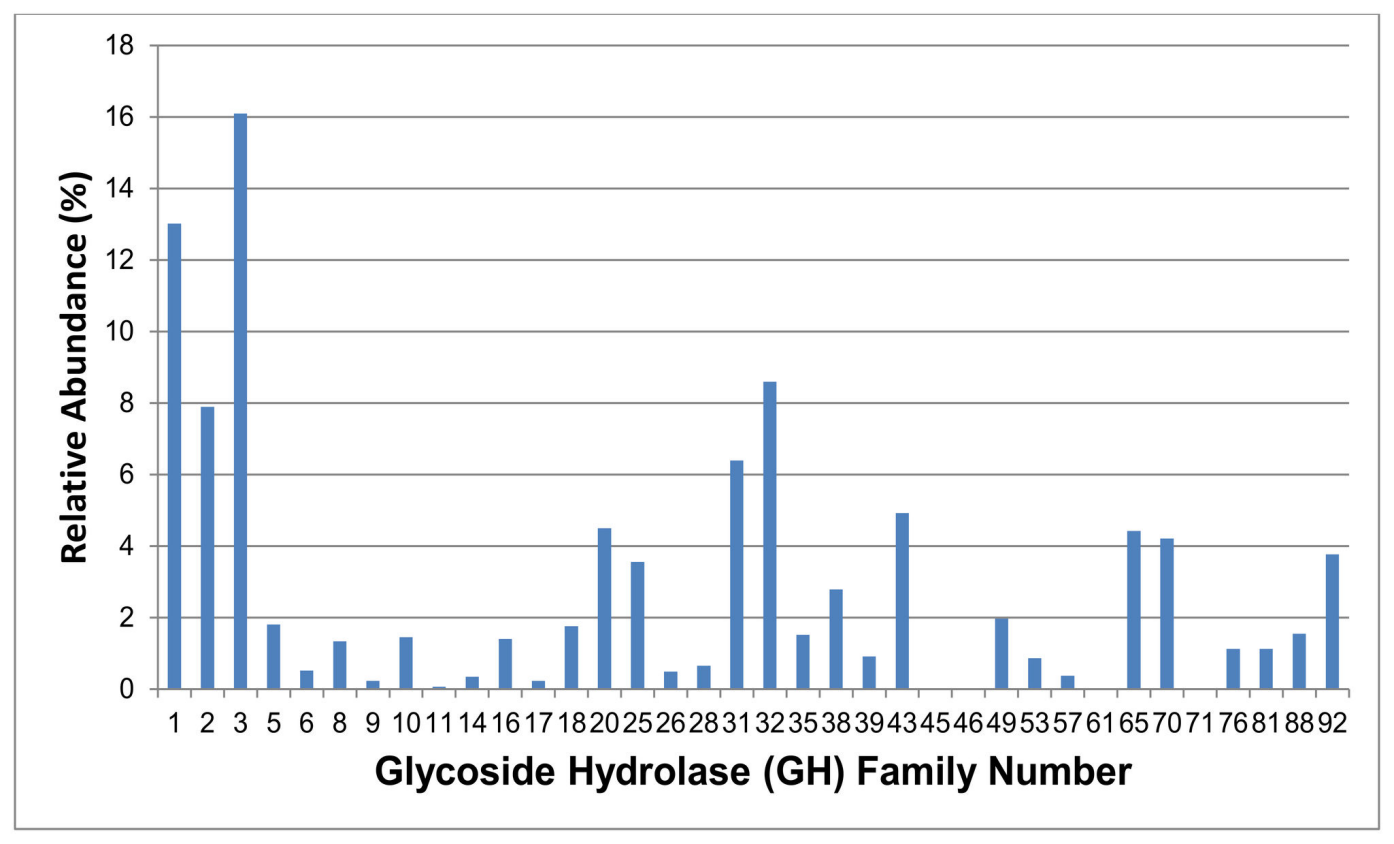
Distribution of glycoside hydrolase families found in the *A* *. glabripennis* gut metagenome. Reads assigned to 36 glycoside hydrolase families were detected in the gut microbiome. The most dominant families were GH 1 and 3, while GH families 11, 45, 46, 61, and 71 were present in very low abundances.

Although many of reads with predicted involvement in carbohydrate digestion are involved in core metabolic pathways, such as glycolysis, many also were annotated by BLAST as accessory enzymes that can digest cellulose and other plant cell wall carbohydrates. For example, reads were classified into 36 different glycoside hydrolase (GH) families based on a combination of Pfam domain and KEGG enzyme class assignments (Figure 7). The most abundant CAZyme (Carbohydrate Active Enzyme) families detected were represented by families GH 1 and GH 3 and their associated KEGG EC assignments are presented in Table 5. The majority of these GH 1 and 3 enzymes were predicted to encode β-glucosidases. KEGG E.C. assignments for all GHs detected in the *A* *. glabripennis* midgut metagenome can be found in Table S2.

**Table 5:** The most highly abundant glycoside hydrolase families detected in gene tag annotations and their associated KEGG classifications.

**GH Family**	**KEGG ECs**	**Reactions**
1,3	β-glucosidase (EC 3.2.1.21)	Hydrolyzes β-1,4 linkages in glucose-containing disaccharides
1	β-galactosidase (EC 3.2.1.23)	Hydrolyzes β-galactosidic bond between galactose and its organic functional group
1	β-mannosidase (EC 3.2.1.25)	Hydrolyzes terminal, non-reducing mannose residues from β-D linked mannosides
1	β-glucuronidase (EC3.2.1.31)	Hydrolyzes β-D glucuronic acid residues from non-reducing end of glycosoaminoglycans
1	Exo-β-1,4-glucanase (EC 3.2.1.74)	Releases cello-oligomers from exposed polysaccharide termini in cellulose
1	6-phospho-β-galactosidase (EC 3.2.1.85)	Hydrolyzes β-galactosidic bond between a 6-phospho-β-D-galactose and its organic functional group
1	6-phospho-β-glucosidase (EC 3.2.1.86)	Hydrolyzes β-1,4 linkages in glucose substituted disaccharides containing phosphorylated glucoside residues
1	Strictosidine amygdalin β-glucosidase (EC 3.2.1.117)	Liberates D-glucose from strictosidine
1	Thioglucosidase (EC 3.2.1.147)	Hydrolyzes linkage between thiol and glycosinolate
1	β-primeverosidase (EC 3.2.1.149)	Hydrolyzes linkage between 6-O-(beta-D-xylopyranosyl)-beta-D-glucopyranoside its organic functional group
3	Xylan 1,4-β -xylosidase (EC 3.2.1.37)	Hydrolyzes linkage between β-linked xylose residues in β-1,4 xylan
3	β -N-acetylhexosaminidase (EC 3.2.1.52)	Liberates hexose from gangliosides
3	Glucan 1,3-β -glucosidase	Cleaves β-1,3 linkages in β glucans
3	Endo-β -1,4-glucanase	Cleaves internal bonds in crystalline cellulose to liberate polysaccharide termini
3	Exo-1,3-1,4-glucanase	Releases cello-oligomers from β-1,3 or β-1,4 linked glucose oligosaccharides and polysaccharides
3	α-L-arabinofuranosidase	Hydrolyszes α-1,3 in arabinose-containing oligosaccharides and polysaccharides

Many of these GH families could have key roles in processing cellulose, hemicellulose, and other plant polysaccharides in the *A* *. glabripennis* midgut. Of particular interest are cellulases (endoglucanases, exoglucanases, and β-glucosidases) that could augment the activities of cellulases inherently produced by *A* *. glabripennis* , enhancing the release of glucose from this highly insoluble and indigestible polysaccharide. Microbial cellulases detected in the *A* *. glabripennis* midgut metagenome were classified to seven different GH families, including GH 1, GH 3, GH 5, GH 6, GH 9, GH 45, and GH 61 and their corresponding KEGG E.C. assignments suggest the presence of all enzymes necessary to liberate glucose from cellulose. We hypothesize that these microbial derived cellulases can collaborate with host enzymes to enhance cellulase activity in the midgut of *A* *. glabripennis* . Alternatively, the overabundance of microbial-derived β-glucosidases may also allow microbes associated with the gut to exploit cellulose degradation products released by endogenous beetle cellulases secreted into the gut; however, the interactions among the beetle and its gut microbes are likely diverse, intricate, and dynamic and an explanation of why these β-glucosidases are overrepresented in this community cannot be fully determined without further investigation. Additionally, reads with highest BLAST scores to components of cellulosomes and other proteins with carbohydrate binding motifs that facilitate binding to the cellulose substrate, allowing hydrolytic enzymes to act processively and efficiently to release cellobiose and other cello-oligomers.

### Candidate Genes for Xylose Utilization and Fermentation

GH families involved in processing hemicellulose were also detected; in general, the structure of hemicellulose is significantly more heterogeneous in comparison to cellulose and is comprised of a matrix of polysaccharides including xylan, glucuronoxylan, arabinoxylan, glucomannan, and xyloglucan. The heterogeneity both in terms of subunit and linkage composition signifies that degrading this prominent group of cell wall polysaccharides requires a greater diversity of enzymes, although xylan and xyloglucans are the dominant hemicellulose polysaccharides in woody plants [82]. Not surprisingly, a number of GH families involved in breaking α- and β-linkages in xylan and xyloglucans were detected in the metagenome, including GH families 5, 8, 10, 11, 26, 39, and 43.

**Figure 8:**
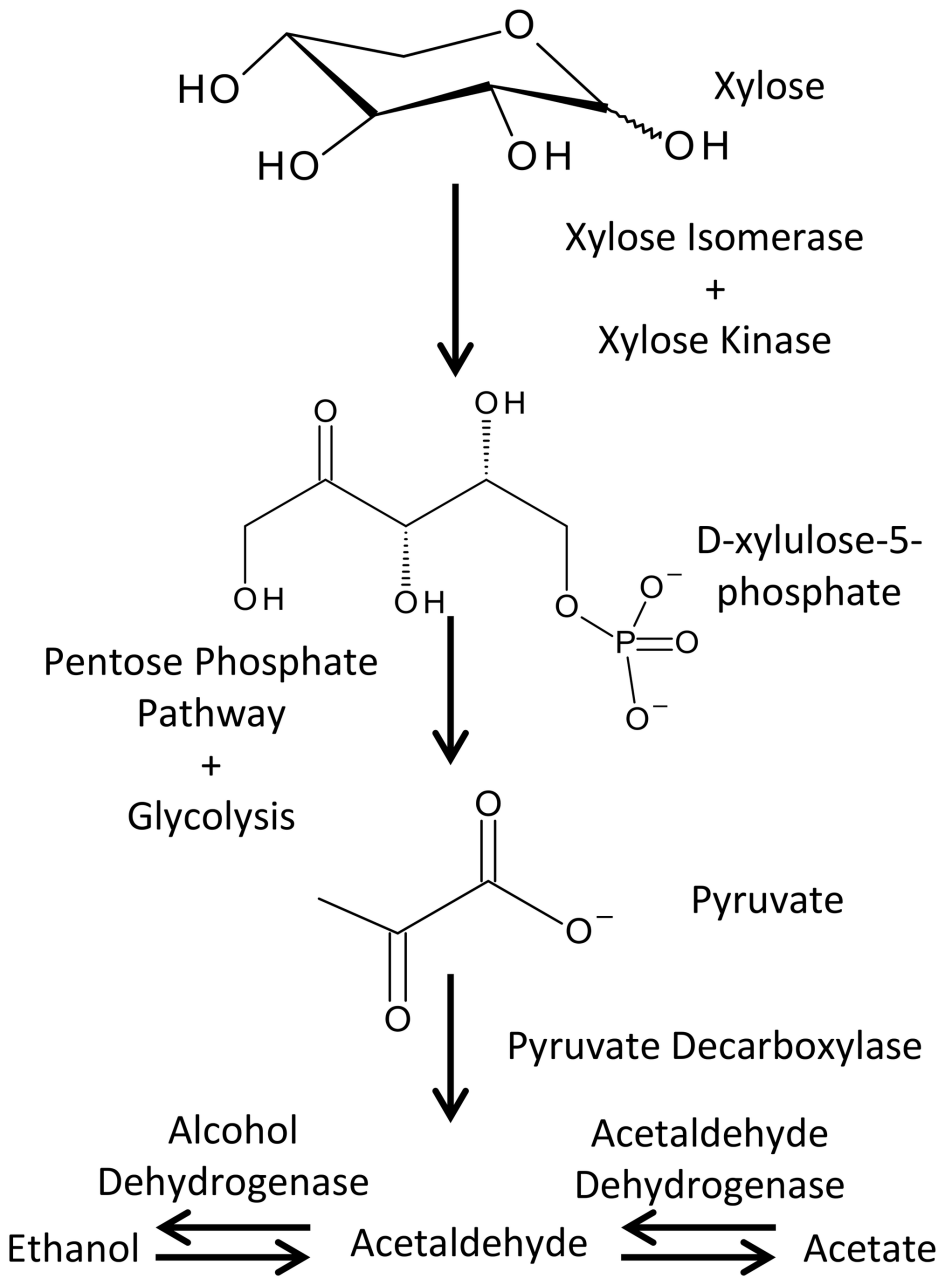
Xylose utilization pathway present in the *A* *. glabripennis* gut community. Xylose released from hemicellulose can be converted into D-xylulose-5-phosphate and eventually into acetaldehyde. Acetaldehyde can be either converted into ethanol by alcohol dehydrogenase or into acetate by acetaldehyde dehydrogenase. These reactions are likely catalyzed by lactic acid bacteria or yeasts associated with the *A* *. glabripennis* gut.

Sugar monomers liberated from xylan can be efficiently metabolized by the midgut microbiota (Figure 8). Of particular importance is the ability to process xylose and arabinose as mechanisms for insect utilization of plant-derived pentose sugars have not been reported [28] and these sugars are inherently difficult to ferment on an industrial scale. Enzymes from both bacterial and fungal xylose isomerase pathways are well represented in the shotgun data to convert D-xylose into D-xylulose-5-phosphate [83]. D-xylulose-5-phosphate can be processed via the pentose phosphate pathway to produce glyceraldehyde-3-phosphate and fructose-6-phosphate, which can enter the glycolysis pathway [84]. Ultimately, pyruvate produced through glycolysis can be converted to acetaldehyde by pyruvate decarboxylase [85] and then to ethanol by alcohol dehydrogenase. Alternatively, acetaldehyde can be oxidized to acetate by acetaldehyde dehydrogenase [86], which can be used as the building blocks for fatty acid production. Although arabinose is a minor constituent of hemicellose in woody plants, it can be converted to D-xylulose-5-phosphate by L-arabinose isomerase and L-ribulokinase where it can be further processed by the pentose phosphate and glycolysis pathways to generate fermentable products [87]. All enzymes required to convert xylose and arabinose to ethanol (or acetate) are present in the *A* *. glabripennis* midgut community. Thus, this community could serve as a reservoir for novel enzymes that could be exploited to enhance industrial xylose fermentation.

### Candidate Genes for Pectin Degrading Enzymes

Liberation of sugar monomers from both cellulose and hemicellulose is greatly enhanced when bonds crosslinking these compounds to pectin and lignin are disrupted, releasing polysaccharide termini and promoting easy access by processive hydrolytic enzymes. Pectin is a polysaccharide comprised primarily of α-galacturonic acid residues and it is often esterified to hemicellulosic and cellulosic polysaccharides in heartwood [88]. Degradation of pectin catalyzed by GH 28 polygalacturonases, pectin lyases, pectin esterases, and pectin acetylases and the disruption of ester linkages between pectin and other structural polysaccharides by carboxylesterases, esterases, and acetyl xylan esterases produced by members of the *A* *. glabripennis* midgut community could indirectly facilitate cellulose and hemicellulose digestion by exposing polysaccharide termini to hydrolytic enzymes. Galacturonic acid residues released from this polysaccharide can be used as an energy source by the gut microbial community or *A* *. glabripennis* as microbial pathways involved in processing galactose and galacturonic acid were detected and pathways involved in galactose utilization have been previously described in beetles [89].

### Candidate Genes for Nutrient Acquisition and Synthesis

Nutrients are extremely scarce in the heartwood where the later instars of *A* *. glabripennis* feed. For example, nitrogen is limiting in woody biomass [90] and nitrogen sources originating from plant cell wall proteins are intricately cross-linked with recalcitrant plant cell wall polysaccharides and biopolymers [91], while other dietary components, including fatty acids, sterols, and vitamins are present in extremely low abundances or are absent altogether [25]. Besides the abilities of cerambycid beetles to produce endogenous cellulases and detoxification enzymes [14,16,92], little is known about their endogenous digestive and metabolic capabilities. Despite this, transcriptome profiling of other Coleopterans revealed that beetles have impressive endogenous digestive and metabolic capabilities and produce diverse arrays of cell-wall degrading enzymes [93] and detoxification enzymes [94,95], however, several pathways leading to the synthesis of sterols [96], aromatic amino acids, and branched chain amino acids are blocked at multiple steps [97] and these nutrients must either be acquired from the food source or through interactions with gut microbes. Because these nutrients are scarce in woody tissue, it is hypothesized that microbes associated with wood-feeding beetles can synthesize essential nutrients, facilitate nutrient recovery from woody tissue, and augment endogenous detoxification enzyme activities [25,98–100].

### Candidate Genes for Nitrogen Acquisition

The C:N ratio in the heartwood of hardwood trees can be as high as 1000:1, although plant cell wall proteins cross-linked in the cell wall matrix may serve as a reservoir of protein sources for organisms that live in this habitat. However, there is much debate about whether or not the protein concentrations in woody tissues are high enough to obtain a sufficient amount of nitrogen for *de novo* synthesis of nucleotides and amino acids. Therefore, it is generally hypothesized that insects and microbes colonizing the heartwood have mechanisms in place to acquire and utilize atmospheric nitrogen or have efficient pathways to recycle nitrogenous waste products [90]. Several bacterial nitrogen fixing genes were identified to convert atmospheric nitrogen to ammonia, which could then be assimilated and used by the beetle and other members of the midgut community. As a consequence, ammonium transporters and glutamine synthases, which actively transport ammonia into the cell and subsequently convert ammonia and glutamate into glutamine, are also highly represented in the *A* *. glabripennis* midgut community. In addition, ammonia (a major byproduct of amino acid deamination reactions) [101], urea (a major waste product of amino acid degradation produced by bacteria) and uric acid (a major nitrogenous waste product produced by insects) [102] represent suitable sources of nitrogen that can be recapitulated and recycled through urease, uricase, and allatonin degradation pathways encoded by the midgut community. Overall, reads assigned to recycling pathways were far more abundant than reads assigned to nitrogen fixing pathways; therefore, we hypothesize that that nitrogen recycling might make important contributions to the nitrogen economy in the larval *A* *. glabripennis* midgut community. Alternatively, nitrogen fixation pathways may also be prominent in the *A* *. glabripennis* community, but these bacteria may be more associated with other regions of the gut where oxygen levels are lower (e.g., hindgut), which were not sampled for this study. Furthermore, a wide array of proteinases with broad substrate abilities is associated with the gut community. This array of enzymes has the capacity to degrade plant proteins released from the plant cell wall matrix during active lignocellulose degradation and scavenge nitrogen from xenobiotic substrates, including cyanide, alkaloids [103], and non-protein amino acids (i.e., cyanoamino acids) [104]. Finally, the gut community possesses full or partial pathways for the synthesis of 23 amino acids, including full pathways for the biosynthesis of aromatic amino acids.

### Candidate Genes for Sterol, Vitamin, and Fatty Acid Synthesis

Other nutrients notably missing or present in low abundances in woody tissue include sterols, vitamins, fatty acids, and inorganic ions [25]. Unlike other animals, insects cannot synthesize cholesterol as this pathway is blocked at several steps; thus, they must acquire sterols that can be converted to cholesterol from their feeding substrate [105]. Many wood-feeding insects (e.g., ambrosia beetles) convert ergosterols produced by cultivated fungal symbionts into cholesterol [106], while others actively convert a variety of phytosterols produced by plants into cholesterol [107]. The *F* *. solani* isolate as well as yeasts harbored in the *A* *. glabripennis* gut have the capacity to contribute to the synthesis of cholesterol and, accordingly, a number of ergosterol synthesis genes (e.g., C-22 sterol desaturase, cytochrome P450s, and lanosterol 14 alpha demethylase) assigned to phylum Ascomycota, were detected. Vitamins and other nutrients missing from woody tissue can be produced or efficiently assimilated by the *A* *. glabripennis* gut community. A combination of acetate, produced via conversion of sugar monomers liberated from woody polysaccharides, and coenzyme A, synthesized by microbial constituents, could be used to synthesize acetyl CoA which is the essential building block for fatty acid synthesis [108]. Furthermore, pathways for synthesizing biotin (vitamin B7), coenzyme A folate (vitamin B9), lipoic acid, pyridoxine (vitamin B6), riboflavin (vitamin B2) thiamine (vitamin B1), and ubiquinone (coenzyme Q10) are well represented in the gut community.

### Candidate Genes for Detoxification

Woody plants produce an array of secondary metabolites and digestive enzyme inhibitors in an attempt to restrict insect herbivory and colonization by pathogenic microbes. These compounds often accumulate in the heartwood of the plant [109]. While many insects endogenously produce impressive arrays of detoxification enzymes or have mechanisms to sequester plant toxins, many beetle species directly benefit from detoxification enzymes produced by microbes [110,111]. For example, microbial communities associated with bark beetles feeding in phloem tissue, which serves as a conduit for toxic defensive chemicals, are highly enriched for detoxification genes [112]. The *A* *. glabripennis* midgut microbial community also encodes genes that can mitigate host plant defenses. A number of bacterial and fungal reads with highest scoring BLAST alignments to host plant inducible cytochrome P450s were detected that are known to promiscuously degrade xenobiotic substrates in an oxidoreductive manner [113]. Reads corresponding to enzymes involved in glutathione-mediated detoxification, including glutathione peroxidases, glutathione-S-transferases, and glutathione reductases, were detected in the gut metagenome. The broad substrate specificities of these quintessential detoxification enzymes allow them to act on a wide range of toxic metabolites produced by many species of host trees. Additionally, most plants produce salicylic acid as a defense mediator against pathogens, which induces the production of defensive compounds. Furthermore, salicylic acid and its regulated pathways have indirect roles in anti-herbivory defenses since they can negatively impact symbiotic microbes associated with herbivores. However, the gut community is capable of producing a number of isochorismatase family proteins hypothesized to disrupt the salicylic acid pathway, which uses isochorismate as a key intermediate [114]. A number of salicylate hydratases were found in the *A* *. glabripennis* gut metagenome that could directly destroy salicylic acid to prevent induction of plant defensive pathways.

Metabolism of lignin also releases highly toxic metabolites, which can cause irreversible damage to the peritrophic matrix, digestive enzymes, and gut-associated microbes. While the cytochrome P450 enzymes mentioned previously could aid in the detoxification of these metabolites, other xenobiotic degrading enzymes were detected that could be involved in these processes, including glutathione S-transferases, glutathione S-peroxidases, epoxide hydrolases, aldo-keto reductases, and alcohol dehydrogenases. Further, several enzymes that hypothesized to directly break down small metabolites released from large-scale lignin degradation were detected in the *A* *glabripennis* metagenome and included lignostilbene-α-β-dioxygenases, 1,2 and 3,4 aromatic ring dioxygenases, biphenyl 2,3 dioxygenases, and *ligX*, *ligZ*, *ligY*, *ligW*, and *ligW1*, which have been observed to coordinate the degradation of ferulic acid and other small molecules released from lignin degradation [115]. A number of enzymes that could function as antioxidants were also detected, which may prevent oxidative damage to the midgut or the microbiota from the ingestion of toxic dietary compounds (e.g. tannins) or from oxidative degradation of lignin.

Finally, one of the most common defense mechanisms employed by plants to reduce herbivory is to produce digestive proteinase enzyme inhibitors to restrict an organism’s ability to break down and assimilate nitrogen [116]. These proteinase enzyme inhibitors typically show high specificity and target a single family of proteinases; however, many insects have evolved a mechanism to overcome these plant defenses by producing a different type of peptidase whose activity and integrity is not impacted by these plant inhibitors [117]. The *A* *. glabripennis* microbial gut community has the genetic capacity to produce an assortment of digestive proteinase classes hypothesized to serve as alternative sources of proteinase family activities in the event that host plant proteinase inhibitors disrupt the endogenous proteinase families produced by *A* *. glabripennis* . Reduction of cysteine proteinase activity in western corn rootworm (Coleoptera: *Diabroticavirgifera* *virgifera*) in antibiotic treated insects has been previously reported [118], demonstrating a role for microbial derived proteinases in insect digestive physiology.

### Candidate Genes from *Fusarium*

Filamentous fungi belonging to the *Fusarium* species complex have been observed in association with beetles collected from all US populations and from several species of host trees. Mass spectroscopy based protein identification techniques and *in vitro* enzyme assays of an *F* *. solani* strain associated with the *A* *. glabripennis* gut cultivated on wood chips demonstrated that this isolate is capable of producing several extracellular laccase enzymes, indicating that this isolate associated with *A* *. glabripennis* has lignin degrading potential. Furthermore, this isolate expressed 28 families of glycoside hydrolases, many of which had predicted cellulase and xylanase activities [62]. In addition to these previously reported findings, genes classified to the genera *Fusarium* /*Nectria* were detected in this analysis included flavin-containing amine oxidoreductases (ammonium generation), glutathione-dependent formaldehyde-activating enzyme (methane metabolism), several sugar transporters, and several short chain dehydrogenases, which can participate in many biochemical processes including sterol synthesis, metabolism of sugar alcohols, and metabolism of fermentation products. Whole genome sequencing is currently underway to compile a complete genetic inventory of this unique fungal strain and will provide a more comprehensive insight into its role in the *A* *. glabripennis* midgut.

### Candidate Genes from *Leuconostoc*

Although sequencing coverage was not deep enough to generate draft genomes of any individual OTU in the *A* *. glabripennis* gut community, roughly 22,000 high quality reads (7.8 Mb) classified to genus *Leuconostoc* were detected in the *A* *. glabripennis* gut metagenome. Bacteria from the genus *Leuconostoc* and other lactic acid bacteria have been previously identified in the guts of *A* *. glabripennis* larvae collected from other populations [9] and several other species of coleopterans (e.g., *Agrilusplanipennis* and beetles in the family Carabidae) [119]. Many genes taxonomically classified to this genus had highest scoring BLAST alignments to xylose fermentation pathways, pathways for utilization of pentose wood sugars, nitrogen recycling enzymes, nutrient synthesizing enzymes, and enzymes with detoxification abilities. A large number of cellobiose phosphorylases and glycoside hydrolase family 1 β-glucosidases were identified, which could be involved in degrading cellobiose disaccharides released from cellulose chains. In addition, a number of genes predicted to encode xylose transporters and xylose fermentation pathways were detected. Further, genes for the uptake and fermentation of other pentose sugars present in hemicellulose, including ribose and arabinose, were detected. Genes annotated as aromatic acid dioxygenases and aryl alcohol dehydrogenases, which could catalyze the degradation of aromatic subunits released from the lignin biopolymer or serve as helper enzymes for β-aryl ether cleavage catalyzed by dyp-type peroxidases, were also identified. Additionally, pathways involved in nutrient synthesis were also detected, which included pathways for the synthesis of branched chain amino acids, aromatic amino acids, sterols, and vitamins as well as enzymes that could function as antioxidants or in detoxification (e.g. cyanide hydratases). Due to the metabolic capacities for pentose sugar fermentation, nutrient synthesis, and detoxification, complete genome assembly for the *Leuconostoc* strains found in association with the *A* *. glabripennis* midgut and more in-depth studies to characterize the interactions between *Leuconstoc* and *A* *. glabripennis* species would be of value to pursue in future research.

## Conclusions

This study represents the first large scale functional metagenomic analysis of the midgut microbial community of a cerambycid beetle with documented lignin degrading capabilities [8]. A taxonomically diverse assemblage of bacteria and fungi are associated with the midgut of *A* *. glabripennis* and this study has shown that this community harbors the enzymatic capacity for extensive contributions to the digestion of woody tissue in this system. Of relevance is i) a microbial community dominated by bacterial and fungal aerobes and facultative anaerobes, indicating an appropriate aerobic environment in the midgut for microbial enzymes involved in oxygen-dependent lignin degradative processes, ii) the similarity of the *A* *. glabripennis* midgut microbiota to the *Sirex* fungal gallery community and its distinction from other herbivore gut communities, including the termite hindgut communities, iii) detection of genes encoding secreted oxidative enzymes proposed to disrupt β-aryl ether linkages and hypothesized to have roles in cleaving β-aryl ether linkages in lignin, iv) detection of extracellular H_2_O_2_-generating enzymes, and v) detection of a number of genera with predicted lignocellulolytic and hemicellulolytic capabilities. The midgut community of *A* *. glabripennis* has the metabolic potential to produce enzymes to help this wood-boring insect overcome major nutritional challenges associated with feeding in woody tissue and we hypothesize that interactions between the beetle and its gut microbes drive this insect’s ability to colonize and thrive in a broad range of healthy host trees. This wood-degrading system should also have great potential for the development of novel lignocellulose degrading enzymes for applications by the biofuels industry. This study provides the first glimpse into the metabolic potential of the gut community associated with a cerambycid beetle and lays the foundations for future hypothesis-based research, including more in-depth biochemical studies, comparative metagenomics, metatranscriptomics, and pathway modeling to assess potential metabolic cross-talk between this beetle and its gut microbes.

## Supporting Information

Figure S1 **MEGAN classification of shotgun reads.** Taxonomic assignments for highly abundant classes (>0.04% relative abundance) detected in the shotgun data. Percentages indicate relative abundance of reads assigned to each class.
(TIF)

Table S1 **Estimated copy number of candidate lignin degrading Pfam domains in herbivore-associated microbial communities.** Estimated copies of Pfam domains detected in each herbivore-related metagenome assembly obtained from IMG/M. To obtain abundances, assembled contigs were multipled by read depth when assembly information was available and singleton reads were treated as single copies.
(XLSX)

Table S2 **Abundance and Class Level Taxonomic Classification of GH Families in the *A* *. glabripennis* gut metagenome.** Corresponding KEGG Enzyme Classifications and class level assignments are also presented.
(DOCX)
