# Supplementary figures and images for: Metagenomic Profiling Reveals Lignocellulose Degrading System in a Microbial Community Associated with a Wood-Feeding Beetle
Source: PLoS One. 2013 Sep 4;8(9):e73827. doi: 10.1371/journal.pone.0073827 (PMC3762729; doi:10.1371/journal.pone.0073827)

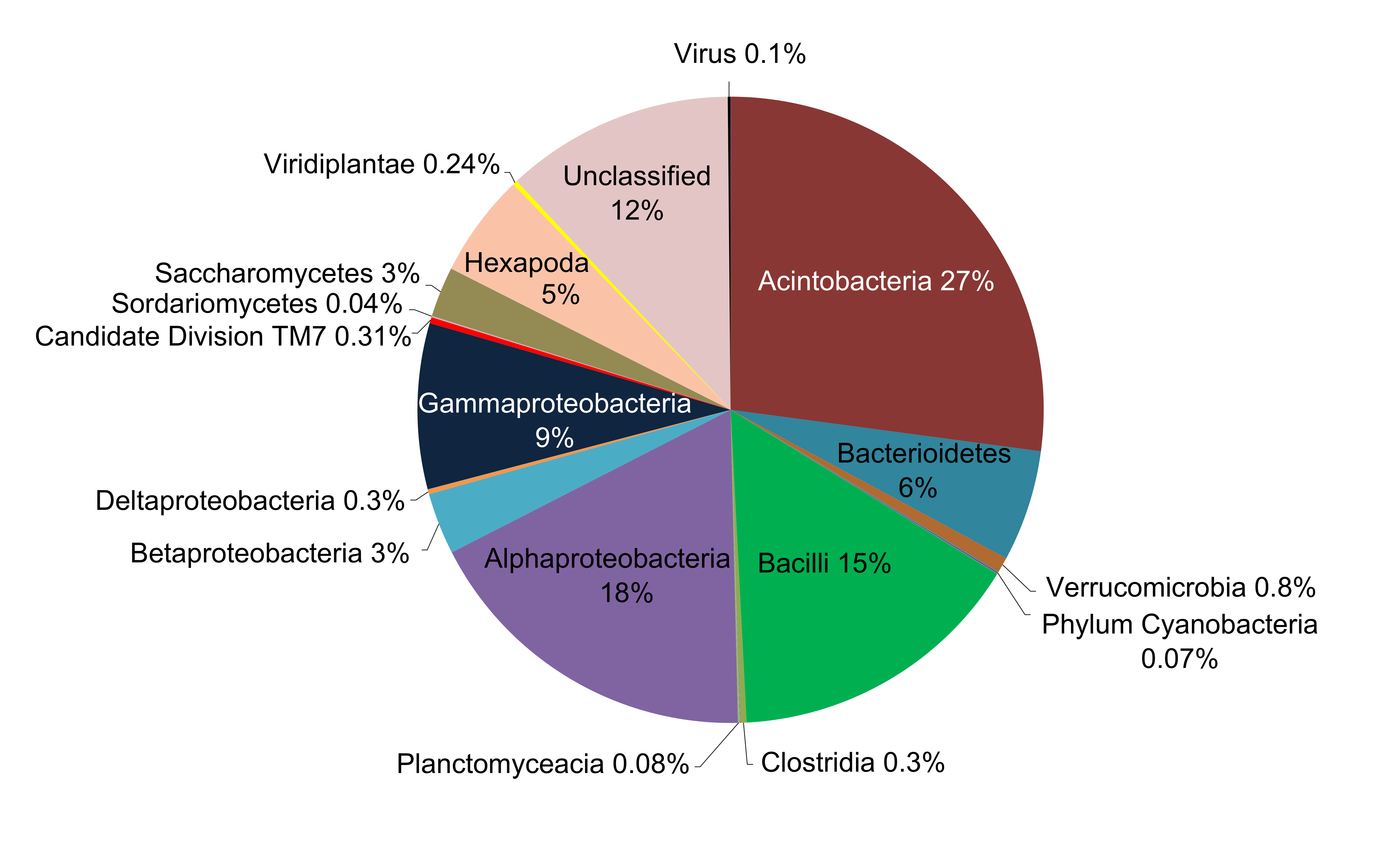

Supplement: Figure S1 — MEGAN classification of shotgun reads. Taxonomic assignments for highly abundant classes (>0.04% relative abundance) detected in the shotgun data. Percentages indicate relative abundance of reads assigned to each class. (TIF) [file pone.0073827.s001.tif]
