# Supplementary material for: Metagenomic Profiling Reveals Lignocellulose Degrading System in a Microbial Community Associated with a Wood-Feeding Beetle
Source: PLoS One. 2013 Sep 4;8(9):e73827. doi: 10.1371/journal.pone.0073827 (PMC3762729; doi:10.1371/journal.pone.0073827)
Supplement: Table S2 — Abundance and Class Level Taxonomic Classification of GH Families in the A . glabripennis gut metagenome. Corresponding KEGG Enzyme Classifications and class level assignments are also presented. (DOCX) [file pone.0073827.s003.docx]

| GH Family  **Table S2. Abundance of GH Families in the *A. glabripennis* gut metagenome.** Corresponding KEGG Enzyme Classifications and class level assignments are also presented. | Number of Reads | KEGG ECs | Class Level Assignments |
| --- | --- | --- | --- |
| 1 | 556 | β-glucosidase (EC 3.2.1.21)  β-galactosidase (EC 3.2.1.23)  β-mannosidase (EC 3.2.1.25)  β-glucuronidase (EC 3.2.1.31)  Exo-β-1,4-glucanase (EC 3.2.1.74)  6-phospho-β-galactosidase (EC 3.2.1.85  6-phospho-β-glucosidase (EC 3.2.1.86)  Strictosidine amygdalin β-glucosidase (EC 3.2.1.117)  Thioglucosidase (EC 3.2.1.147)  β-primeverosidase (EC 3.2.1.149) | Actinobacteria  Alphaproteobacteria  Bacilli  Bacteroidetes  Betaproteobacteria  Clostridia  Gammaproteobacteria  Hexapoda  Saccharomycetes  Verrucomicrobia |
| 2 | 337 | β-galactosidase (EC 3.2.1.23)  β-mannosidase (EC 3.2.1.25)  β-glucuronidase (EC 3.2.1.31)  Mannosylglycoprotein | Actinobacteria  Alphaproteobacteria  Bacilli  Bacteroidetes  Gammaproteobacteria  Hexapoda  Lentisphaeria |
| 3 | 687 | β-glucosidase (EC 3.2.1.21)  Xylan 1,4-β -xylosidase (EC 3.2.1.37)  β -N-acetylhexosaminidase (EC 3.2.1.52)  Glucan 1,3-β -glucosidase (EC 3.2.1.58)  Endo-β -1,4-glucanase (EC 3.2.1.74)  Exo-1,3-1,4-glucanase (EC 3.2.1.-)  α-L-arabinofuranosidase (EC 3.2.1.55) | Acidobacteria  Actinobacteria  Alphaproteobacteria  Bacilli  Bacteroidetes  Betaproteobacteria  Clostridia  Gammaproteobacteria  Lentisphaeria  Coleoptera  Saccharomycetes  Thermobaculum  Verrucomicrobia |
| 5 | 77 | β-mannosidase (EC 3.2.1.25)  Endo- β -1,4-glucanase (EC 3.2.1.4)  Glucan β -1,3-glucosidase (EC 3.2.1.58)  Licheninase (EC 3.2.1.73)  Glucan endo-1,6- β -glucosidase (EC 3.2.1.75)  Mannan endo-β-1,4-mannosidase (EC 3.2.1.78)  Endo- β -1,4-xylanase (EC 3.2.1.8)  Exo-β-1,4-cellobiosidase (EC 3.2.1.91)  β -1,3-mannanase (EC 3.2.1.-)  Mannan transglycosylase (EC 2.4.1.-)  β -glucosylceramidase (EC 3.2.1.45) | Actinobacteria  Alphaproteobacteria  Bacilli  Bacteroidetes  Clostridia  Gammaproteobacteria  Hexapoda  Lentisphaeria  Verrucomicrobia |
| 6 | 22 | Endo-β -1,4-glucanase (EC 3.2.1.4)  Exo-β -1,4-cellobiosidase (EC 3.2.1.91) | Actinobacteria  Alphaproteobacteria  Bacilli  Bacteroidetes  Chloroflexi  Clostridia  Gammaproteobacteria  Hexapoda  Saccharomycetes |
| 8 | 57 | Endo-β -1,4-glucanase (EC 3.2.1.4)  Licheninase (EC 3.2.1.73)  Endo-1,4-β-xylanase (EC 3.2.1.8) | Actinobacteria  Alphaproteobacteria  Bacilli  Gammaproteobacteria |
| 9 | 10 | Endo-β -1,4-glucanase (EC 3.2.1.4)  Exo-β -1,4-cellobiosidase (EC 3.2.1.91)  β-glucosidase (EC 3.2.1.21); | Actinobacteria Alphaproteobacteria  Bacilli  Bacteroidetes Gammaproteobacteria  Deltaproteobacteria |
| 10 | 62 | Endo-1,4-β-xylanase (EC 3.2.1.8)  Endo-1,3-β-xylanase (EC 3.2.1.32) | Actinobacteria  Alphaproteobactia  Bacteroidetes  Gammaproteobacteria  Verrucomicrobia |
| 11 | 3 | Endo-1,4-β-xylanase (EC 3.2.1.8) | Actinobacteria |
| 14 | 15 | β-amylase (EC 3.2.1.2) | Actinobacteria  Bacteroidetes  Verrucomicrobia |
| 15 | 92 | Glucoamylase (EC 3.2.1.3)  Glucodextranase (EC 3.2.1.70)  α,α-trehalase (EC 3.2.1.28) | Actinobacteria  Alphaproteobacteria  Bacteroidetes  Gammaproteobacteria  Hexapoda  Saccharomycetes |
| 16 | 60 | Endo-1,3-β-glucanase (EC 3.2.1.39)  Endo-1,3(4)-β-glucanase (EC 3.2.1.6  Licheninase (EC 3.2.1.73)  Endo-β-1,3-galactanase (EC 3.2.1.-) | Actinobacteria  Alphaproteobacteria  Bacteroidetes  Gammaproteobacteria  Verrucomicrobia |
| 17 | 10 | Endo-1,3-β-glucosidase (EC 3.2.1.39)  Glucan 1,3-β-glucosidase (EC 3.2.1.58)  Licheninase (EC 3.2.1.73)  β -1,3-glucanosyltransglycosylase (EC 2.4.1.-) | Saccharomycetes |
| 18 | 75 | Chitinase (EC 3.2.1.14) | Actinobacteria  Bacteroidetes  Clostridia  Gammaproteobacteria  Hexapoda  Saccharomycetes  Verrucomicrobia |
| 20 | 192 | β-hexosaminidase (EC 3.2.1.52)  β -1,6-N-acetylglucosaminidase (EC 3.2.1.-)  β -6-SO3-N-acetylglucosaminidase (EC 3.2.1.-) | Actinobacteria  Alphaproteobacteria  Bacteroidetes  Betaproteobacteria  Gammaproteobacteria  Hexapoda  Lentisphaeria |
| 25 | 152 | Lysozyme (EC 3.2.1.17) | Actinobacteria  Alphaproteobacteria  Bacilli  Bacteroidetes  Gammaproteobacteria |
| 26 | 21 | β-mannanase (EC 3.2.1.78) | Actinobacteria  Alphaproteobacteria  Bacilli  Bacteroidetes  Gammaproteobacteria  Saccharomycetes |
| 28 | 26 | Polygalacturonase (EC 3.2.1.15)  Exo-polygalacturonase (EC 3.2.1.67)  Endo-xylogalacturonan hydrolase (EC 3.2.1.-) | Actinobacteria  Alphaproteobacteria  Bacilli  Bacteroidetes  Gammaproteobacteria  Saccharomycetes |
| 31 | 273 | α-glucosidase (EC 3.2.1.20)  α -1,3-glucosidase (EC 3.2.1.84)  Sucrase-isomaltase (EC 3.2.1.48) Isomaltosyltransferase (EC 2.4.1.-) | Actinobacteria  Alphaproteobacteria  Bacilli  Bacteroidetes  Clostridia  Gammaproteobacteria  Hexapoda  Lentisphaeria  Saccharomycetes  Verrucomicrobia |
| 32 | 367 | Invertase (EC 3.2.1.26)  Endo-levanase (EC 3.2.1.65)  Sucrose:fructan 6-fructosyltransferase (EC 2.4.1.10)  Levan fructosyltransferase (EC 2.4.1.-) | Actinobacteria  Alphaproteobacteria  Bacilli  Bacteroidetes  Betaproteobacteria  Gammaproteobacteria  Saccharomycetes |
| 35 | 65 | β-galactosidase (EC 3.2.1.23)  Exo-β-1,4-galactanase (EC 3.2.1.-) | Actinobacteria  Bacteroidetes  Hexapoda  Verrucomicrobia |
| 38 | 119 | α-mannosidase (EC 3.2.1.24)  Mannosyl-oligosaccharide-α -1,3-mannosidase (EC 3.2.1.-) | Acidobacteria  Actinobacteria  Alphaproteobacteria  Bacilli  Bacteroidetes  Clostridia  Lentisphaeria  Hexapoda  Saccharomycetes  Thermobaculum |
| 39 | 39 | β-xylosidase (EC 3.2.1.37) | Actinobacteria  Alphaproteobacteria  Lentisphaeria  Hexapoda  Verrucomicrobia |
| 43 | 210 | β -xylosidase (EC 3.2.1.37)  β -1,3-xylosidase (EC 3.2.1.-)  α-L-arabinofuranosidase (EC 3.2.1.55)  Arabinanase (EC 3.2.1.99)  Endo-1,4-β-xylanase (EC 3.2.1.8) | Actinobacteria  Bacilli  Bacteroidetes  Clostridia  Gammaproteobacteria |
| 45 | 1 | Endo-β -1,4-glucanase (EC 3.2.1.4) | N/A* |
| 46 | 1 | No EC evidence | Bacilli |
| 47 | 20 | α-mannosidase (EC 3.2.1.113) | Hexapoda  Saccharomycetes |
| 51 | 84 | Dextranase (EC 3.2.1.11) | Actinobacteria |
| 53 | 37 | Endo-β-1,4-galactanase (EC 3.2.1.89) | Actinobacteria  Alphaproteobacteria  Bacteroidetes  candidate division TM7  Gammaproteobacteria |
| 57 | 16 | α-amylase (EC 3.2.1.1)  4- α-glucanotransferase (EC 2.4.1.25)  α--galactosidase (EC 3.2.1.22)  amylopullulanase (EC 3.2.1.41) | Actinobacteria  Alphaproteobacteria  Bacteroidetes  candidate division TM7  Gammaproteobacteria |
| 61 | 1 | Copper-dependent polysaccharide monooxygenases | N/A* |
| 65 | 189 | α,α-trehalase (EC 3.2.1.28)  Maltose phosphorylase (EC 2.4.1.8)  Kojibiose phosphorylase (EC 2.4.1.230)  Trehalose-6-phosphate phosphorylase (EC 2.4.1.-) Nigerose phosphorylase (EC 2.4.1.-) | Actinobacteria  Bacilli  Bacteroidetes  Clostridia  Saccharomycetes |
| 67 | 10 | α-glucuronidase (EC 3.2.1.139) | Actinobacteria  Alphaproteobacteria  Bacteroidetes  Gammaproteobacteria |
| 70 | 180 | Dextransucrase (EC 2.4.1.5)  Reuteransucrase (EC 2.4.1.-)  α-4,6-Glucanotransferase (EC 2.4.1.-) | Actinobacteria  Alphaproteobacteria  Bacilli  Bacteroidetes |
| 71 | 1 | No EC evidence | Actinobacteria |
| 76 | 48 | α-1,6-mannanase (EC 3.2.1.101) | Actinobacteria  Alphaproteobacteria  Bacteroidetes  Saccharomycetes |
| 81 | 48 | Endo-β-1,3-glucanase (EC 3.2.1.39) | Actinobacteria  Bacteroidetes  Saccharomycetes |
| 85 | 4 | No EC evidence | Actinobacteria |
| 88 | 66 | D-4,5-unsaturated-β-glucuronyl hydrolase (EC 3.2.1.-) | Actinobacteria  Alphaproteobacteria  Bacilli  Bacteroidetes  Gammaproteobacteria  Hexapoda  Verrucomicrobia |
| 92 | 161 | Mannosyl-oligosaccharide-α-1,2-mannosidase (EC 3.2.1.113)  Mannosyl-oligosaccharide-α -1,3-mannosidase (EC 3.2.1.-)  Mannosyl-oligosaccharide-α-1,6-mannosidase (EC 3.2.1.-)  α -mannosidase (EC 3.2.1.24)  α -1,2-mannosidase (EC 3.2.1.-)  α -1,3-mannosidase (EC 3.2.1.-)  α -1,4-mannosidase (EC 3.2.1.-) | Actinobacteria  Bacilli  Bacteroidetes  Gammaproteobacteria |
